# Quality of medicines for life-threatening pregnancy complications in low- and middle-income countries: A systematic review

**DOI:** 10.1371/journal.pone.0236060

**Published:** 2020-07-10

**Authors:** Maria Regina Torloni, Mercedes Bonet, Ana Pilar Betrán, Carolina C. Ribeiro-do-Valle, Mariana Widmer

**Affiliations:** 1 Evidence Based Healthcare Post-Graduate Program, Sao Paulo Federal University, Sao Paulo, SP, Brazil; 2 UNDP/UNFPA/UNICEF/WHO/World Bank Special Programme of Research, Development and Research Training in Human Reproduction (HRP), Department of Sexual and Reproductive Health and Research, World Health Organization, Geneva, Switzerland; 3 Department of Obstetrics Gynecology, University of Campinas (UNICAMP) School of Medical Sciences, Campinas, SP, Brazil; Eberhard-Karls-Universitaet Tuebingen, GERMANY

## Abstract

**Background:**

There are concerns about the quality of medicines available in low- and middle-income countries (LMIC) to manage hemorrhage, pre-eclampsia/eclampsia and sepsis. We aimed to identify, critically appraise, and synthesize the findings of studies on the quality of these three types of medicines available in LMIC.

**Methods:**

This systematic review searched Medline, EMBASE and LILACS (from inception to 25 May 2020) for studies on the quality of selected medicines available in LMIC that provided at least the amount of active pharmaceutical ingredient. We contacted study authors for additional information. We excluded simulation studies. We used the MEDQUARG tool to assess study quality. The main outcome was the prevalence of failed samples.

**Findings:**

We identified 9699 unique citations and included 34 studies (3159 samples from 40 countries) in the review. Most studies (65%) had low quality (scores <6/12). Overall, 48.9% of 1890 uterotonic samples (19 studies) failed quality tests; failures rates were 75% for ergometrine and nearly 40% each for oxytocin and misoprostol. The overall prevalence of failed injectable antibiotics (1090 samples, 18 studies) was 13.4%, ranging from 2.9% for injectable metronidazole (34 samples, 3 studies) to 16.0% for cefazolin (449 samples, 2 studies). The prevalence of low quality magnesium sulphate (179 samples, 2 studies) was 3.4%. We did not find any studies on the quality of carbetocin, tranexamic acid, or clindamycin.

**Conclusions:**

There is a widespread problem with the quality of medicines used to manage life-threatening maternal conditions in LMIC. This can be a contributing factor to high maternal mortality rates in these regions.

## Introduction

An estimated 295,000 women die every year due to complications of pregnancy and childbirth.[1] Most of these deaths occur in low- and middle-income countries (LMIC) and could be avoided with adequate healthcare.[2, 3] Postpartum hemorrhage (PPH), pre-eclampsia/eclampsia (PE/E), and sepsis due to direct maternal infections cause 27%, 14% and 11% of all maternal deaths, respectively [2–4] and are also major contributors to severe maternal morbidity.[5]

The adequate and timely use of good quality, safe, effective, and affordable medicines is essential to achieve universal health coverage and reduce maternal morbidity and mortality[5]. The World Health Organization (WHO) recommends the use of uterotonics/antifibrinolytics, magnesium sulphate, and antibiotics to manage PPH, PE/E, and maternal infection/sepsis, respectively.[6–8] These medications are part of the WHO Essential Medicines List.[9]

Substandard and falsified (SF) medicines are a worldwide health problem that can harm patients and populations.[10] According to the latest official definition, substandard medicines are “authorized drugs that fail to meet either their quality standards or specification, or both”.[10] Substandard medicines, also known as “out of specification” drugs, can be the result of poor manufacturing, shipping, or storage conditions, or the sale of a product after its expiration date. Falsified medicines are defined as “medical products that deliberately/fraudulently misrepresent their identity, composition or source”.[10]

It is estimated that 1 in 10 medicines in LMIC are SF and there are growing concerns about their potential negative health impact.[11–15] In 2016, we conducted a systematic review on the quality of oxytocin in LMIC and reported that 46% of the samples failed quality tests.[16] There are several reports on the quality of medicines used to manage life-threatening pregnancy complications in LMIC [17–23] but no systematic reviews on this specific topic. This motivated us to update our previous systematic review on the quality of oxytocin and to expand our evidence synthesis to other medicines used for PPH, PE/E and maternal sepsis in LMIC.

The objectives of this systematic review were to identify, critically appraise, and synthesize the findings of studies on the quality of selected medicines available in LMIC recommended for use in healthcare facilities to manage pregnancy complications leading to the main causes of maternal death.

## Methods

This systematic review followed the Meta-analysis Of Observational Studies in Epidemiology (MOOSE) framework [24] and was reported according to the Preferred Reporting Items for Systematic Reviews and Meta-Analyses (PRISMA) statement.[25] The protocol of this review was not registered. The process of screening, study selection and quality assessment was conducted in duplicate, by two independent reviewers. Discrepancies were discussed until agreement was reached, with the participation of a third reviewer if needed.

### Search strategy and selection criteria

#### Types of studies

We included published or unpublished studies/reports that assessed the quality of selected medicines collected in LMIC. The assessment of quality of a medicine includes several aspects such as visual inspection of the product (packaging and product insert), expiration date, and laboratory testing for compliance with pharmacopoeial standards including the presence and quantity of the active pharmaceutical ingredient (API), sterility, presence of impurities, and bioavailability data.[26] Medicine potency (defined as the amount of API in a product) is the most frequently reported parameter in field studies since it is directly linked to product effectiveness. We included only studies that assessed at least the amount of API of any of the medicines in our list. Studies that assessed any number of samples, collected in the public or private (including non-governmental organizations) sectors, and in central (warehouses, central stores) or peripheral (pharmacies, hospitals, clinics, informal market vendors) level outlets were eligible. We included studies published only as abstracts if they provided sufficient information. We excluded studies reporting only physical inspection/packaging of medicines without content analysis, studies without information on the tests performed or the number of samples tested and failed, studies that only compared analytical laboratory methods, and simulation studies that investigated the effects of external conditions on the quality of medicines.

#### Types of medicines

We included medicines recommended by WHO for the management of PPH, PE/E and direct maternal infections/sepsis.[7–9] We selected 12 medicines: five for PPH (carbetocin, ergometrine, misoprostol, oxytocin and tranexamic acid), one for PE/E (magnesium sulphate) and six parenteral antibiotics (ampicillin, cefazolin, clindamycin, gentamycin, metronidazole, penicillin G).

### Study identification and selection

The search strategy was created with the assistance of an experienced librarian and run in EMBASE, Medline, and LILACS, from inception to 6 April, 2019, without language or publication status restrictions (S1 Appendix). The search was run again on 25 May, 2020. We screened the reference lists of articles selected for full text reading and relevant systematic reviews, and contacted investigators who had conducted/were conducting studies on the quality of medicines to identify additional potentially relevant reports.

Citations retrieved from electronic databases were uploaded into Covidence (Veritas Health Innovation, Melbourne, Australia) and duplicates were excluded. Titles and abstracts were screened, and full texts of potentially relevant studies were obtained. Studies that fulfilled the aforementioned selection criteria were included.

### Data extraction and quality assessment

Reviewers used a data extraction form created for this review to collect the following information from each study: date and place (country, sector and type of outlet) of sample collection, country of manufacture, number of sample assessed, tests performed and results. We assessed the risk of bias of each study using the MEDQUARG (Medicine Quality Assessment Reporting Guidelines) checklist [27] adapted by Almuzaini [11] to assess 12 methodological aspects of each included study. Studies with total scores ≥ 6 (maximum 12) were considered of good methodological quality.[11]

### Data synthesis and statistical analyses

We present the prevalence of failed samples (failure rate) for each medicine in each study according to the primary study authors´ definition of failure, if available. If this was not defined, we considered samples with 90–110% of the labelled amount of the product as being of adequate quality.[26] For studies that performed several simultaneous quality tests on the same sample, we accepted the parameters used by primary study authors to classify samples as being of adequate or inadequate quality. For example, if samples with API 90–110% were judged by primary study authors to be of inadequate quality because they were not compliant with other parameters (such as sterility), we also classified these as failed samples. We collected the number of samples without any API or the wrong API, when provided by study authors, as a possible indication of falsified medicine.

We classified the countries where studies were conducted as low-income (LIC), lower-middle-income (LrMIC) or upper-middle-income countries (UMIC) according to the World Bank.[28] We report and compare the average prevalence of failed samples for each of the three major groups of medicines. When available, we compared average failure rates per type of sector (private, including non-governmental organizations, versus public) and outlet level (central versus peripheral) where samples were collected. We used Chi square and two-sided Fisher exact tests to compare average failure rates between groups. P < 0.05 was considered significant. We did not pool the prevalence of failed samples into metanalyses because of differences between studies in the definitions of failure, sampling and testing methods, and small sample sizes.[27] Therefore, we did not assess how the risk of bias of individual studies could affect the cumulative evidence.

## Results

**Fig 1:**
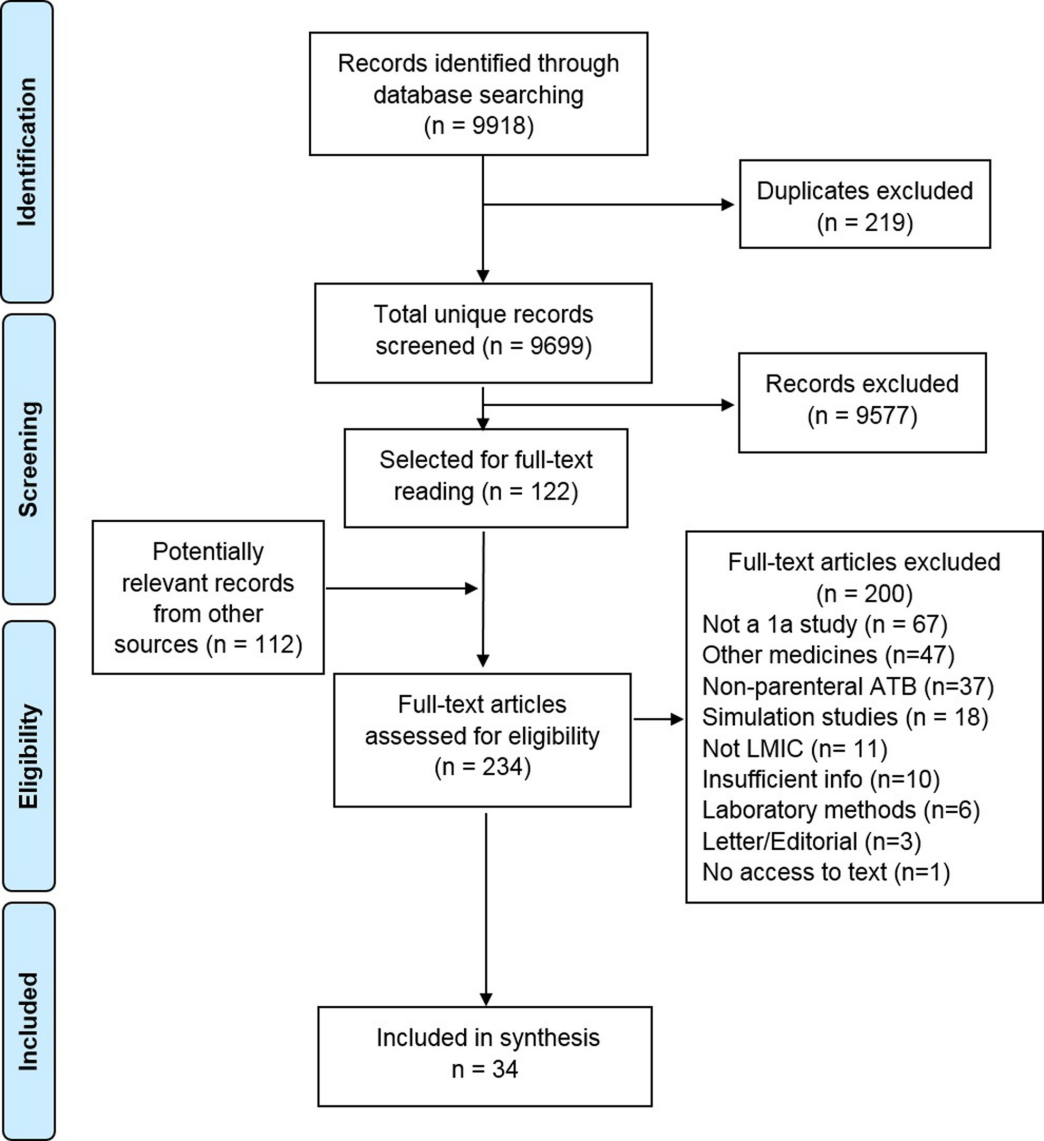
Flow chart of the process of study identification and selection. ATB: antibiotics, LMIC: Low- and middle-income countries.

A total of 9699 unique citations were identified from electronic databases. After initial screening, 122 studies were selected for full text reading, along with 112 reports from other sources. We excluded 200 (S2 Appendix) and included 34 studies in the review [17, 19–21, 23, 29–57] (Fig 1). Two of the studies were congress abstracts [40, 46], 11 were reports [23, 29, 30, 34, 36, 37, 41–43, 48, 55], one was a pre-publication manuscript [44] and 20 were studies published in journals.

The 34 studies collected a total of 3159 samples from 40 different countries, mostly located in Africa (N = 14) and Asia (N = 12) (Tables 1 and S1). Most studies (N = 18) collected samples only in LrMIC. Nearly half of the studies (N = 15) collected samples after 2011. Samples were collected in the private and public sectors, and central as well as peripheral level outlets. Over one third of the studies (N = 12) did not have information on the country where the medicines were manufactured. Over 70% (n = 24) of the studies assessed other quality aspects besides API (S1 Table).

**Table 1:** Main characteristics of 34 studies on quality of medicines for maternal health from low- and middle-income countries.

Characteristic	N Studies	References
**Region**		
Africa	14	17, 19, 21, 31, 34, 36, 38, 39, 43, 51, 52, 53, 55, 56
Asia	12	20, 29, 32, 35, 37, 40, 44, 45, 46, 47, 48, 50,
Americas	4	30, 41, 42, 49
Oceania	1	57
>1 region	3	23, 33, 54
**Country Income Level**^1^		
Low	6	19, 29, 37, 38, 39, 56
Lower-middle	18	17, 20, 21, 31, 35, 36, 42, 43, 44, 45, 46, 47, 48, 50, 51, 52, 53, 57
Upper-middle	4	30, 32, 41, 49
>1 income level	6	23, 33, 34, 40, 54, 55
**Year of sample collection**		
Up to 2000	7	33, 43, 45, 52, 53, 54, 55
2001–2011	12	31, 32, 42, 37, 41, 46, 30, 48, 49, 50, 51,21
2012 and after	15	17, 56, 33, 35, 19, 36, 40, 38, 39, 29, 44, 47, 57, 20, 23
**Number of medicines assessed**		
1	22	19, 20, 29, 30, 32, 33, 35, 37, 38, 39, 40, 41, 42, 44, 45, 46, 47, 48, 49, 52, 54, 55
2	5	34, 36, 50, 51, 56
3	5	17, 31, 43, 53, 57
5	2	21, 23
**Total number of samples analyzed**		
10 or less	6	30, 31, 41, 42, 45, 53
11–99	18	19, 21, 29, 34, 35, 37, 38, 39, 40, 44, 47, 48, 49, 52, 54, 55, 56, 57
100 or more	10	17, 20, 23, 32, 33, 36, 43, 46, 50, 51
**Sector of sample collection**		
Private only	5	20, 38, 45, 48, 50
Public only	5	29, 34, 43, 46, 49
Private and Public	14	17, 19, 23, 30, 33, 35, 36, 37, 39, 41, 42, 51, 55,56
No information	10	21, 31, 32, 40, 44, 47, 52, 53, 54, 57
**Level of sample collection**^2^		
Central level only	2	23,57
Peripheral level only	14	19, 20, 30, 37, 38, 40, 41, 45, 48, 49, 50, 51, 52, 54
Central and Peripheral	15	17, 29, 31, 33, 34, 35, 36, 39, 42, 43, 44, 46, 47, 55, 56
No information	3	21, 32, 53
**Place of manufacture**		
National	4	32, 43, 46, 50
Imported	10	17, 42, 34, 36, 38, 39, 45,47, 52, 56
National and Imported	8	20, 21, 23, 30, 31, 35, 41, 53
No information	12	19, 29, 33, 37, 40, 44, 48, 49, 51, 54, 55, 57
**Quality of study** ^3^		
Low	22	19, 21, 23, 29, 30, 31, 32, 33, 34, 35, 37, 40, 42, 43, 41, 45, 47, 49, 52, 53, 54, 55
High	12	17, 20, 32, 36, 38, 39, 44, 46, 48, 50, 51, 56, 57

1. According to World Bank https://datahelpdesk.worldbank.org/knowledgebase/articles/906519-world-bank-country-and-lending-groups.

2. Central level: warehouses, major distributors or central medical stores. Peripheral level: clinics, hospitals, local medical stores, pharmacies, or markets that sell directly to costumers

3. Quality scores on MEDQUARG 12 domains checklist: Low: total score < 6 points, High: total score ≥ 6 (Almuzaini 2013)

The 34 studies assessed a total of 3159 samples of nine of the 12 medicines in our list. We did not find any study on carbetocin, tranexamic acid, or clindamycin. Nineteen studies assessed the quality of 1890 uterotonic samples, two studies assessed 179 samples of magnesium sulphate, and 18 studies assessed 1090 samples of injectable antibiotics (Table 2). In the studies that stratified results per setting of collection, most samples were from the private sector (1106/1865) and from peripheral level outlets (823/2057).

**Table 2:** Types of medicines and number of samples assessed in 34 studies included in systematic review.

Medicine	N of samples	N of Studies	References
**Uterotonics**			
Oxytocin	979	14	17, 23, 34, 36, 38, 39,40, 41, 42, 44, 46, 50, 51, 56
Ergometrine	500	8	19, 31, 34, 36, 43, 50, 51, 54
Misoprostol	411	3	17, 33, 56
Total	1890	19*	17, 19, 23, 31, 33, 34, 36, 38, 39, 40, 41, 42, 43, 44, 46, 50, 51, 54, 56
**Anticonvulsant**			
Magnesium sulphate	179	2	17, 23
**Injectable Antibiotics**			
Ampicillin	266	7	20, 21, 23, 29, 43, 49, 57
Cefazolin	449	2	21, 32
Gentamycin	223	9	21, 23, 30, 31, 35, 37, 47, 48, 53
Metronidazole	34	3	21, 53, 57
Penicillin G	118	9	21, 23, 31, 43, 45, 52, 53, 55, 57
Total	1090	18*	20, 21, 23, 29, 30, 31, 32, 35, 37, 43, 45, 47, 48, 49, 52, 53, 55, 57
**TOTAL**	**3159**	**34**	

* Several studies assessed >1 uterotonic or >1 antibiotic

Approximately 35% (N = 12) of the 34 studies were considered of good methodological quality (scores ≥ 6) (Tables 1 and S2). Final quality scores ranged from 1 to 11 (maximum: 12). Almost all studies scored highly on the domains related to the description of the chemical analyses performed (domain 10) and type of outlet sampled (domain 3). The domains related to the description of statistical analyses (domain 9), blinding of chemical assessors (domain 12) and details on method validation (domain 11) had the lowest scores. Less than 30% (N = 10) of the studies reported that they had used random sampling (S2 Table). The proportion of good quality studies increased over time, from 0% in the studies that collected samples prior to 2001 to 53.3% (P = 0.054, Fischer´s exact test) in those that collected samples in 2012 or after (S1 Fig).

**Fig 2:**
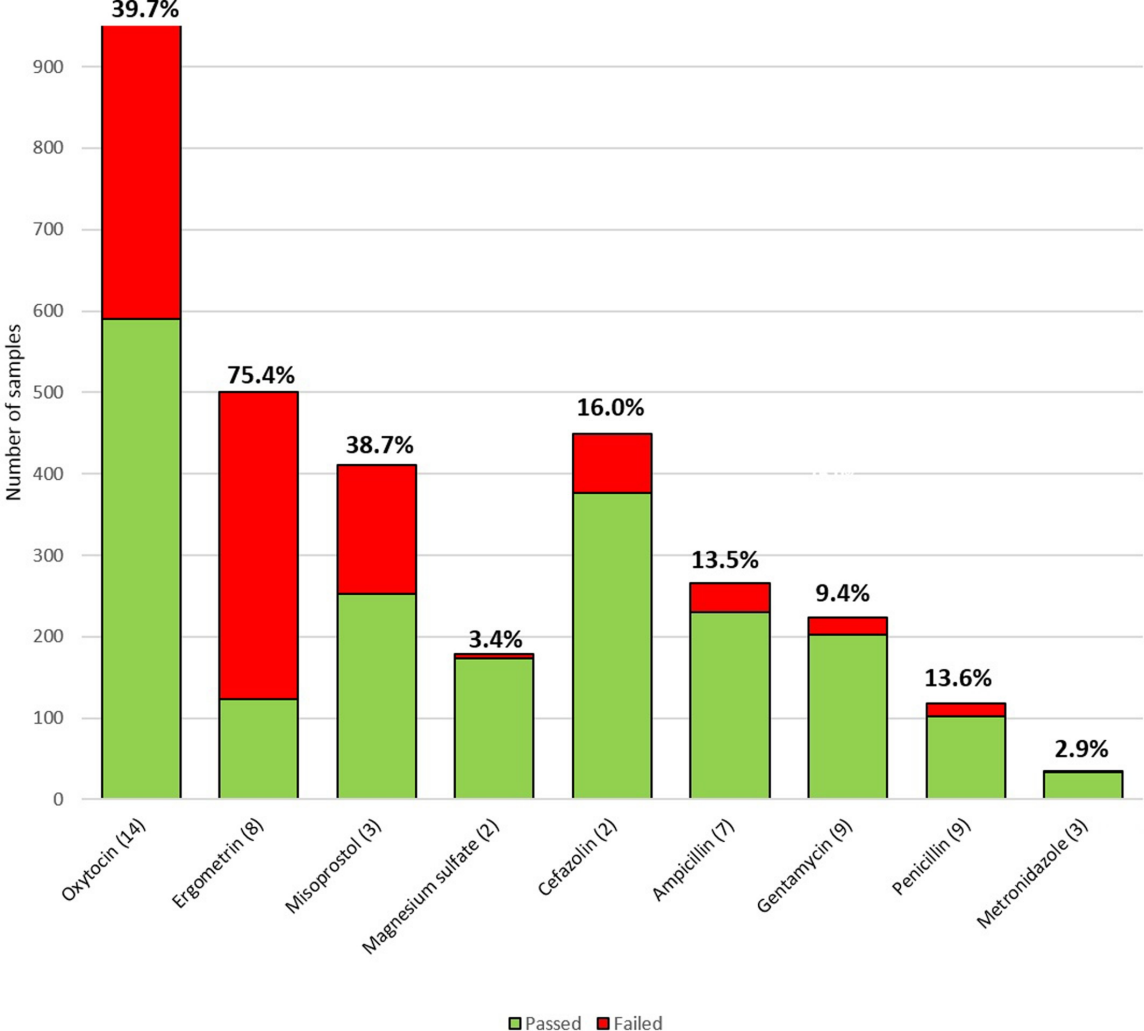
Prevalence of failed medicines used to manage treat life-threatening maternal conditions in LMIC. * Prevalence of failed samples for each medicine (number of failed samples/ total number of samples assessed). ** Number in parentheses indicates the number of studies for each medicine.

Nearly half (48.9%, range 0–100%, 19 studies) of 1890 uterotonic samples failed quality assessments. The prevalence of failed samples was highest for ergometrine (75.4%, range 0–100%, 8 studies), followed by oxytocin (39.7%, range 0–80%, 14 studies) and misoprostol (38.7%, range 23.3–44.7%, 3 studies) (Table 3 and Fig 2). For the three uterotonics assessed together, the prevalence of failed samples was significantly higher in the private than in the public sector (54.3% vs 45.0%, P = 0.001) and in peripheral than in central level outlets (52.7% vs 33.9%, P<0.001). The prevalence of failed oxytocin samples was significantly higher in the private sector than in the public sector (48.7% x 34.3%, p<0.001), and in peripheral than in central level outlets (43.8% vs 21.9%, P<0.001). There were no statistically significant differences in the prevalence of low quality ergometrine or misoprostol samples collected in public versus private sectors, or in central versus peripheral level outlets (Table 3). One ergometrine, four oxytocin, and 15 misoprostol samples had no active ingredients, possibly corresponding to falsified medicines (S1 Table). The prevalence of failed uterotonic samples decreased significantly over time, from 70.1% for the 147 samples collected up to 2000, to 50.1% for the 607 samples collected in 2001–2011, reaching 45.6% in the 1136 samples collected after 2011 (P<0.001) (S3 Table). There was a significant decline in the proportion of failed oxytocin samples over time (80% vs 31.4% or 44.4% in samples collected before 2001, in 2001–2011 and after 2011, respectively, p<0.001). The prevalence of failed ergometrine samples did not differ significantly over time (69.7% vs 77.9% vs 72.2%, samples collected before 2001, in 2001–2011 and after 2011, respectively). We could not conduct this comparison for misoprostol because all sample were collected after 2011. (S3 Table). Uteronic fails were due to inadequate amounts of API (S1 and S4 Tables). Low API was the main problem; in the studies that provided this information, the API content was mostly between 76% to 89% of the declared API content (S4 Table).

**Table 3:** Prevalence of uterotonic samples that failed quality tests.

Study	Country	All samples			Public sector samples			Private sector samples			Central level^1^ samples			Facility level^2^ samples		
		Total	Fails		Total	Fails		Total	Fails		Total	Fails		Total	Fails	
		N	n	(%)	N	n	(%)	N	n	(%)	N	n	(%)	N	n	(%)
**Oxytocin**																
Stanton 2012	Ghana	46	35	(76.1)	NI	NI	NI	NI	NI	NI	0	-	-	46	35	(76.1)
Karikari 2013	Ghana	169	94	(55.6)	90	48	(53.3)	79	46	(58.2)	7	3	(42.9)	162	91	(56.2)
Stanton 2014	India	193	69	(35.8)	0	-	-	193	69	(35.8)	0	-	-	193	69	(35.8)
Hogerzeil 1993	Zimbabwe	5	4	(80.0)	5	4	(80.0)	0	-	-	0	-	-	5	4	(80.0)
Pribluda 2012	Indonesia	110	10	(9.1)	110	10	(9.1)	0	-	-	19	0	(0.0)	91	10	(11.0)
MQ Database 2011	Guatemala	6	0	(0.0)	5	0	(0.0)	1	0	(0.0)	2	0	(0.0)	4	0	(0.0)
MQ Database 2010	Peru	8	0	(0.0)	6	0	(0.0)	2	0	(0.0)	0	-	-	8	0	(0.0)
UNCol LSC	10 countries^3^	22	8	(36.4)	9	2	(22.2)	13	6	(46.2)	22	8	(36.4)	0	-	-
Anyakora 2018	Nigeria	159	118	(74.2)	49	39	(79.6)	110	79	(71.8)	8	5	(62.5)	151	113	(74.8)
Lambert 2018	DR Congo	15	12	(80.0)	NI	NI	NI	NI	NI	NI	0	-	-	15	12	(80.0)
PATH 2015	India	94	14	(14.9)	NI	NI	NI	NI	NI	NI	NI	NI	NI	NI	NI	NI
Lambert 2019	Ethiopia	45	2	(4.4)	32	2	(6.3)	13	0	(0.0)	3	0	(0.0)	42	2	(4.8)
Liu 2016	Nepal. Vietnam	42	13	(31.0)	NI	NI	NI	NI	NI	NI	0	-	-	42	13	(31.0)
Hagen 2020	Malawi	65	10	(15.4)	NI	NI	NI	NI	NI	NI	12	3	(25.0)	53	7	(13.2)
**Total**		**979**	**389**	**(39.7)**	**306**	**105**	**(34.3)**^**5**^	**411**	**200**	**(48.7)**^**5**^	**73**	**16**	**(21.9)** ^**5**^	**812**	**356**	**(43.8)** ^**5**^
**Ergometrin**																
Stanton 2012	Ghana	55	55	(100.0)	NI	NI	NI	NI	NI	NI	0	-	-	55	55	(100.0)
Karikari 2013	Ghana	99	73	(73.7)	36	23	(63.9)	59	46	(78.0)	3	2	66.7	92	67	(72.8)
Stanton 2014	India	188	135	(71.8)	0	-	-	188	135	(71.8)	0	0	0	188	135	(71.8)
Hozergeil 1993	Malawi. Gambia. Sudan. Zimbabwe	25	19	(76.0)	NI	NI	NI	NI	NI	NI	5	3	(60.0)	20	16	(80.0)
Kaale 2016	Tanzania	15	15	(100.0)	12	12	(100.0)	3	3	100.0	0	0	0	15	15	(100.0)
Walker 1988	Bangladesh. DR Yemen. Zimbabwe	24	15	(62.5)	NI	NI	NI	NI	NI	NI	0	0	0	24	15	(62.5)
Nazerali 1996	Zimbabwe	93	65	(69.9)	93	65	(69.9)	0	-	-	26	17	(65.4)	67	48	(71.6)
Abuga 2013	Kenya	1	0	(0.0)	NI	NI	NI	NI	NI	NI	NI	NI	NI	NI	NI	NI
**Total**		**500**	**377**	**(75.4)**	**141**	**100**	**(70.9)**	**250**	**184**	**(73.6)**	**34**	**22**	**(64.7)**	**461**	**351**	**(76.1)**
**Misoprostol**																
Anyakora 2018	Nigeria	166	56	(33.7)	55	21	(38,2)	111	35	(31.5)	5	0	(0.0)	161	56	(34.8)
Hall 2016	15 countries^4^	215	96	(44.7)	NI	NI	NI	NI	NI	NI	NI	NI	NI	NI	NI	NI
Hagen 2020	Malawi	30	7	(23.3)	NI	NI	NI	NI	NI	NI	6	2	(33.3)	24	5	(20.8)
**Total**		**411**	**159**	**(38.7)**	**55**	**21**	**(38.2)**	**111**	**35**	**(31.5)**	**11**	**2**	**(18.2)**	**185**	**61**	**(33.0)**
**ALL UTEROTONICS**		**1890**	**925**	**(48.9)**	**502**	**226**	**(45.0)** ^**5**^	**772**	**419**	**(54.3)** ^**5**^	**118**	**40**	**(33.9)** ^**5**^	**1458**	**768**	**(52.7)** ^**5**^

NI: No information.

1. Central level: warehouses, major distributors or central medical stores.

2. Peripheral level: clinics, hospitals, local medical stores, pharmacies, or markets that sell directly to costumers.

3. UNCol 10 countries: Burkina Faso, Kenya, Madagascar, Nepal, Nigeria, Tajikistan, Tanzania, Uganda, Vietnam, Zimbabwe.

4. Hall 2016 15 countries: Argentina, Bangladesh, Cambodia, Egypt, India, Indonesia, Mexico, Kazakhstan, Kenya, Nepal, Nigeria, Pakistan, Peru, Vietnam, Philippines.

5. P <0.001.

Two studies assessed 179 magnesium sulphate samples. The overall prevalence of failed samples was 3.4% (range 2.5–10.5%) (Table 4 and Fig 2). The prevalence of failure was significantly higher in the 60 samples collected in the public than in the 119 collected in the private sector (8.3% vs 0.8%, P = 0.017). There were no significant differences in the quality of samples collected in central versus peripheral level outlets (Table 4). We could not analyse changes in the prevalence of failed samples over time because both studies that assessed the quality of this medicine were conducted after 2011 (S3 Table). Two magnesium sulphate samples failed due to pH problems (S1 Table). The other four failed samples had inadequate API but the studies did not provide details on how severely the content deviated from specifications (S4 Table).

**Table 4:** Prevalence of magnesium sulphate samples that failed quality tests.

Study	Country	All samples			Public sector samples			Private sector samples			Central level^1^ samples			Peripheral level^2^ samples		
		Total	Fails		Total	Fails		Total	Fails		Total	Fails		Total	Fails	
		N	n	(%)	N	n	(%)	N	n	(%)	N	n	(%)	N	n	(%)
**Magnesium sulphate**																
Anyakora 2018	Nigeria	160	4	(2.5)	53	3	(5.7)	107	1	(0.9)	11	0	(0.0)	149	4	(2.7)
UNCol 2015	10 countries^3^	19	2	(10.5)	7	2	(28.6)	12	0	(0.0)	19	2	(10.5)	0	-	-
**TOTAL**		179	6	(3.4)	60	5	(8.3)^4^	119	1	(0.8)^4^	30	2	(6,7)	149	4	(2.7)

1. Central level: warehouses, major distributors or central medical stores.

2. Peripheral level: clinics, hospitals, local medical stores, pharmacies, or markets that sell directly to costumers.

3. UNCol 10 countries: Burkina Faso, Kenya, Madagascar, Nepal, Nigeria, Tajikistan, Tanzania, Uganda, Vietnam, Zimbabwe.

4. P = 0.017

The overall prevalence of failed injectable antibiotic samples was 13.4% (range 0% to 55%) (Table 5). Failure rate was lowest for metronidazole (2.9%, range 0% to 50%, 3 studies) and highest for cefazolin (16.0%, range 0% to 16.1%, 2 studies) (Table 5 and Fig 2). For the five injectable antibiotics assessed together, failure rates were significantly higher in the private than the public sector (20.3% vs 8.1%, p = 0.001) but did not differ significantly in peripheral versus central level outlets. Seven studies assessed the quality of 266 ampicillin samples in LMIC with an overall failure rate of 13.5%. Failure rates were significantly higher in the private than the public sector (21.5% vs 8.8%, P = 0.007), without significant differences in peripheral versus central level outlets. Only two studies assessed the quality of cefazolin, mostly in China, and reported an overall failure rate of 16.0% without data on type of sector or outlet. The overall prevalence of low quality gentamycin was 9.4%, without significant differences in the samples collected in the public and private sectors, but significantly higher in those collected in central than in peripheral level outlets (19.4% vs 5.6%, P = 0.009). Based on data from three small studies the overall failure rate for injectable metronidazole was 2.9%, without data on type of sector or outlet. The overall failure rate of 118 penicillin G samples from seven studies was 13.6%, without significant differences between types of sectors, but a significantly higher prevalence of failed samples in facility than in central outlets (20.6% vs 0%, p = 0.004) (Table 5). One penicillin G and three gentamycin samples did not have any active ingredients (S1 Table). There was a significant decline in the proportion of failed antibiotic samples over time, (21.2% vs 14.2% vs 10.3% in samples collected before 2001, in 2001–2011 and after 2011, respectively, p = 0.006) (S3 Table). The main reason for failed samples was inadequate API, but most studies did not provide details on the exact API deviation (S4 Table).

**Table 5:** Prevalence of injectable antibiotics that failed quality tests.

Study	Country	All samples			Public sector samples			Private sector samples			Central level^1^ samples			Facility level^2^ samples		
		Total	Fails		Total	Fails		Total	Fails		Total	Fails		Total	Fails	
		N	n	(%)	N	n	(%)	N	n	(%)	N	n	(%)	N	n	(%)
**Ampicillin**																
UnCol 2015	10 countries^3^	26^4^	9	(34.6)	9	3	(33.3)	17	6	(35.3)	26	9	(34.6)	0	-	-
Silva 2010	Brazil	13	0	(0.0)	13	0	(0.0)	0	-	-	0	-	-	13	0	(0.0)
Nazerali 1996	Zimbabwe	34	7	(20.6)	34	7	(20.6)	0	-	-	10	2	(20.0)	24	5	(20.8)
Tabernero 2019	Laos	104	20	(19.2)	0	-	-	104	20	(19.2)	0	-	-	104	20	(19.2)
Thoithi 2008	Kenya	2	0	(0.0)	NI	NI	NI	NI	NI	NI	NI	NI	NI	NI	NI	NI
Afghanistan 2015	Afghanistan	57^4^	0	(0.0)	57	0	(0.0)	0	-	-	NI	NI	NI	NI	NI	NI
Scrimgeour 2019	Papua New Guinea, Vanatu, Solomon Islands	30	0	(0,0)	NI	NI	NI	NI	NI	NI	30	0	(0,0)	0		
TOTAL		266	36	(13.5)	113	10	(8.8)^5^	121	26	(21.5)^5^	66	11	(16.7)	141	25	(17.7)
**Cefazolin**																
Dan Ling 2013	China	447	72	(16.1)	NI	NI	NI	NI	NI	NI	NI	NI	NI	NI	NI	NI
Thoithi 2008	Kenya	2	0	(0.0)	NI	NI	NI	NI	NI	NI	NI	NI	NI	NI	NI	NI
TOTAL		449	72	(16.0)												
**Gentamycin**																
UnCol 2015	10 countries^3^	29	12	(41.4)	9	4	(44.4)	20	8	(40.0)	29	12	(41.4)	0		
Islam 2018	Myanmar	58	3	(5.2)	NI	NI	NI	NI	NI	NI	NI	NI	NI	NI	NI	NI
Rafiqul Islam 2017	Cambodja	59	0	(0.0)	NI	NI	NI	NI	NI	NI	33	0	(0.0)	26	0	(0.0)
Sheth 2007	India	20	2	(10.0)	0	-	-	20	2	(10.0)	0	-	-	20	2	(10.0)
Thoithi 2008	Kenya	3^4^	0	(0.0)	NI	NI	NI	NI	NI	NI	NI	NI	NI	NI	NI	NI
Thoithi 2002	Kenya	3^4^	1	(33.3)	NI	NI	NI	NI	NI	NI	NI	NI	NI	NI	NI	NI
Abuga 2013	Kenya	8	0	(0.0)	NI	NI	NI	NI	NI	NI	NI	NI	NI	NI	NI	NI
Karwar 2011	Afghanistan	35	0	(0.0)	19	0	(0.0)	16	0	(0.0)	0	-	-	35	0	(0.0)
SAIDI-Peru 2009	Peru	8	3	(37.5)	NI	NI	NI	NI	NI	NI	0	-	-	8	3	(37.5)
TOTAL		223	21	(9.4)	28	4	(14.3)	56	10	(17.9)	62	12	(19.4)^5^	89	5	(5.6)^5^
**Metronidazole**																
Thoithi 2008	Kenya	2	0	(0.0)	NI	NI	NI	NI	NI	NI	NI	NI	NI	NI	NI	NI
Thoithi 2002	Kenya	2	1	(50.0)	NI	NI	NI	NI	NI	NI	NI	NI	NI	NI	NI	NI
Scrimgeour 2019	Papua New Guinea, Vanatu, Solomon Islands	30	0	(0,0)	NI	NI	NI	NI	NI	NI	30	0	0,0	0		
TOTAL		34	1	(2.9)							30	0	(0.0)			
**Penicillin**																
UnCol 2015	10 countries^3^	6^4^	0	(0.0)	3	0	(0.0)	3	0	(0.0)	6	0	(0.0)	0	-	-
Taylor 2001	Nigeria	20	11	(55.0)	NI	NI	NI	NI	NI	NI	0	-	-	20	11	(55.0)
Prazuck 2002	Myanmar	2	1	(50.0)	0	-	-	2	1	(50.0)	0	-	-	2	1	(50.0)
WHO 1995	Cameroon, Madagascar, Tchad	14	2	(14.3)	NI	NI	NI	NI	NI	NI	NI	NI	NI	NI	NI	NI
Nazerali 1996	Zimbabwe	41	1	(2.4)	41	1	(2.4)	0	-	-	0	-	-	41	1	(2.4)
Thoithi 2008	Kenya	2^4^	0	(0.0)	NI	NI	NI	NI	NI	NI	NI	NI	NI	NI	NI	NI
Thoithi 2002	Kenya	2^4^	1	(50.0)	NI	NI	NI	NI	NI	NI	NI	NI	NI	NI	NI	NI
Abuga 2013	Kenya	1	0	(0.0)	NI	NI	NI	NI	NI	NI	NI	NI	NI	NI	NI	NI
Scrimgeour 2019	Papua New Guinea, Vanatu, Solomon Islands	30	0	(0,0)	NI	NI	NI	NI	NI	NI	30	0	(0,0)	0		
TOTAL		118	16	(13.6)	44	1	(2.3)	5	1	(20.0)	36	0	(0.0)^5^	63	13	(20.6)^5^
**ALL ANTIBIOTICS**		**1090**	**146**	**(13.4)**	**185**	**15**	**(8.1)**^5^	**182**	**37**	**(20.3)**^5^	**194**	**23**	**(11.9)**	**293**	**43**	**(14.7)**

NI: No information.

1. Central level: warehouses, major distributors or central medical stores.

2. Peripheral level: clinics, hospitals, local medical stores, pharmacies, or markets that sell directly to costumers

3. UNCol 10 countries: Burkina Faso, Kenya, Madagascar, Nepal, Nigeria, Tajikistan, Tanzania, Uganda, Vietnam, Zimbabwe.

4. Samples consisted of powders.

5. P<0.05.

## Discussion

There is a widespread problem with the quality of medicines available in LMIC for the management of PPH, PE/E and maternal sepsis. The problem is more evident for uterotonics (nearly 50% failure rates), and critical for ergometrine (75% failure rates). Similarly, 1 in 7 injectable antibiotic samples (13%) and 1 in 29 magnesium sulphate samples (3.4%) were of low quality. Nearly half of the studies assessed samples collected since 2011, indicating that the quality of these medicines is a current global concern. Although the prevalence of failed uterotonics and antibiotics samples has decreased over time, it is still high.

To our knowledge, apart from oxytocin [16], this is the first systematic review on the quality of medicines available in LMIC used to manage conditions that lead to severe maternal morbidity and mortality. We strived to reduce bias by following strict methods including duplicate study selection, data extraction, and quality assessment. We created a sensitive search strategy and also searched for and found several reports and an unpublished manuscript. However, it is possible that we may have missed relevant (especially unpublished) reports. Additional limitations of the review include the low methodological quality of most included studies, the small number of samples for some medicines, the paucity of samples collected in the Americas and in UMIC, and the lack of important details such as medicine manufacturer, or the period and place of sample collection in several studies. Finally, our findings cannot be generalized as being representative of the overall quality of these medicines available in the countries where they were collected because most included studies did not use random sampling and large sample sizes, as recommended for reliable estimates.[27]

Oxytocin was the drug with the largest number of studies and samples. The six additional studies on oxytocin conducted after the publication of our previous review[16] continue to show substandard quality of this medicine in LMIC, especially in peripheral level outlets. Ergometrine was the uterotonic with the highest prevalence of failed samples. This is not surprising if we consider that this drug has to be stored at 2–8°C and protected from light. Most of the studies that assessed the quality of ergometrine collected the samples at peripheral level where problems maintaining a continuous cold chain are frequently reported. In addition ergometrine is very unstable under the influence of light. [34]. Despite the well know stability problems of ergometrine and the existence of other alternative uterotonics, most of the studies on the quality of this medicine were conducted over the last eight years which suggests that ergometrine is still available and used in LMIC. When issues with the cold chain are detected, people tend to replace oxytocin and ergometrine with misoprostol because it is a drug that is stable at room temperature. However, our review also found quality problems in nearly 40% of the misoprostol samples, and a high number of possibly falsified misoprostol samples. These findings contradict the general perception that misoprostol does not have quality problems and could replace oxytocin in settings without cold-chain conditions.

Clindamycin is an antibiotic in the WHO EML but its availability and cost may be limiting factors to its use in low-resource settings [9, 58]. This may be a reason why we found no studies that assessed the quality of clindamycin in LMIC. Due to the lack of studies on carbetocin and tranexamic acid, which have only recently been added to essential medicines lists, we have no information on potential quality problems for these medicines in LMIC. This is an important gap that will need to be addressed in future studies as tranexamic acid and heat-stable carbetocin have been included in the 2019 WHO Essential Medicines List [6], and will start to be used for PPH in many countries. The low failure rates for magnesium sulphate samples could be attributed to the fact that it has a very simple formulation and is very stable at ambient temperatures. Therefore, magnesium sulphate ampoules are unlikely to undergo any significant degradation if they are properly manufactured, sterilized and packaged.[59]

Although there were substandard medicines in both sectors, in general we found higher failure rates in the private sector. Differences in policies for regulation and procurement of medicines across sectors may contribute to these findings.[13] Medicines prequalified by WHO or registered by a regulator applying similarly stringent requirements are regarded as reliable and of high quality, but these products cost 5%-10% more than medicines without such characteristics.[60] When national procurement bodies or private providers decide not to adhere to these standards in an attempt to lower costs, this can lead to higher expenses and burden due to additional interventions needed to treat complications.[61]

Since most samples from the 34 studies were collected in peripheral level outlets, it is not possible to infer the cause for their low quality. Poor quality medicines can be due to problems related to manufacturing or storage conditions during transport along the supply chain, or at the final point of distribution.[62] Some of the medicines included in this review (e.g. oxytocin, and ergometrine) are sensitive to high temperatures, and others need to be protected from light (e.g. ergometrine) or humidity (e.g. misoprostol). If manufacturers do not follow good manufacturing practices or if storage conditions recommended on product labels are not followed, it is very likely that these medicines will already be of low quality when they leave the warehouse, or will suffer degradation during transport and storage.

Substandard and falsified medicines can have negative individual and public health impacts beyond those related to the management of life-threatening maternal conditions. For instance, low quality oxytocin can affect labour induction or augmentation, falsified misoprostol can contribute to failed inductions, and substandard magnesium sulphate can compromise the effectiveness of fetal neuroprotection protocols. Overall, low-quality medicines could increase mortality, morbidity, and lead to additional costs.[15, 62] Poor quality antimicrobials can contribute to the spread of antimicrobial resistance, a global health concern.[63] Substandard medicines can also negatively affect the confidence of the general public and healthcare professionals in medical products and health systems, and lead to actions such as overdosing.[64] In a survey conducted in Nigeria, over half of 705 healthcare providers reported to have administered more than the recommended dose of oxytocin to prevent PPH, a possible reflection of their perception of the lack of potency of the medicine available.[64] The arbitrary decision to administer higher doses of oxytocin because of concerns of drug potency is potentially dangerous and can expose women to unnecessary risks.[65]

As countries move towards achieving sustainable development goals and universal health coverage, greater attention should be given to quality of medicines.[61, 66] Improving access to high-quality medicines in LMIC requires action from many stakeholders.[62] Governmental and non-governmental organizations should prioritize improvements in the quality of medicines that are essential to save mothers’ lives.[13] Efforts should focus on regulations that encourage the use medicines with WHO prequalification or approved by regulators that use equally stringent requirements. Countries also need to invest in human and technical capacity to ensure that quality and safety of medicines are monitored effectively and continuously by government agencies, starting when drugs are manufactured until they are administered to the patients.

More studies are needed on the quality of magnesium sulphate, injectable metronidazole, clindamycin, carbetocin, and tranexamic acid available in LMIC. Future studies should also assess the quality of medicines used for PPH, PE/E and maternal sepsis in upper middle-income countries.

## Conclusions

There is a widespread problem with the quality of medicines used in LMIC to manage life-threatening maternal conditions. This could be a contributing factor to the persistence of maternal deaths due to PPH, PE/E, and sepsis in these settings, despite affordable and effective treatments.

## Supporting information

S1 Appendix Search strategy. (DOCX)

S2 Appendix List of 200 excluded studies with reasons. (DOCX)

S1 Checklist PRISMA check list. (DOCX)

S1 Fig Quality scores of 34 studies over time. (DOCX)

S1 Table Main details of 34 studies included in systematic review. (DOCX)

S2 Table Quality scores of 34 studies included in systematic review. (DOCX)

S3 Table Prevalence of failed samples over time. (DOCX)

S4 Table Failed samples due to inadequate API. (DOCX)
