# Supplementary material for: Quality of medicines for life-threatening pregnancy complications in low- and middle-income countries: A systematic review
Source: PLoS One. 2020 Jul 10;15(7):e0236060. doi: 10.1371/journal.pone.0236060 (PMC7351160; doi:10.1371/journal.pone.0236060)
Supplement: S1 Appendix — (DOCX) [file pone.0236060.s001.docx]

**S1 Appendix. Search Strategy**

| # | EMBASE (via Ovid) |
| --- | --- |
| 1 | (uterotonic agent) or (uterotonic agents).ab,hw,kf,ot,rn,sh,sy,ti,nm,mp. |
| 2 | \|  \| exp Oxytocin/ or ('orasthin' or ' orastina ' or ' oxitocin ' or ' oxiton inj ' or ' oxitone ' or ' oxystin ' or ' oxytan ' or ' oxytocina ' or ' oxytocine ' or ' pareton ' or ' partacon ' or ' partocon ' or ' partolact ' or ' partoxin ' or ' physormon ' or ' pitocin ' or ' piton ' or ' pituilobine ' or ' pitupartin ' or ' solvoxine ' or ' synpitan ' or ' syntocinon ' or ' tranoxy ' or ' utedrin ' or ' uteracon ' or ' uterason ' or ' utron ' or ' xitocin ').ab,hw,kf,ot,rn,sh,sy,ti,nm,mp. \| \| --- \| --- \| |
| 3 | (carbetocina' or crinesal' or duratobal' or duratocin' or pabal').ab,hw,kf,ot,rn,sh,sy,ti,nm,mp. |
| 4 | \|  \| Exp ergometrine/ \| \| --- \| --- \| |
| 5 | (8beta ergometrine' or 'd lysergic acid 1 ydroxymethylethylamide' or 'd lysergic acid l 2 propanolamide' or 'ergobasine' or 'ergoklinine' or 'ergometrin' or 'ergonovine' or 'ergostetrine' or ergotocine' or 'ergotrate' or 'hydroxypropyllysergamide' or 'lysergic acid propanolamide').ab,hw,kf,ot,rn,sh,sy,ti,nm,mp. |
| 6 | \|  \| exp 'tranexamic acid'/ or (' aminomethyl cyclohexanecarboxylic ' or ' 4 aminomethylcyclohexanecarbonic acid ' or ' 4 aminomethylcyclohexanecarboxylic acid ' or ' amca ' or ' AMCHA ' or ' amchafibrin ' or ' amikapron ' or ' amino methylcyclohexane* ' or ' aminomethyl cyclohexane ' or ' aminomethyl cyclohexane* ' or ' minomethylcyclohexanecarboxylic* ' or ' amstat ' or ' anexan ' or ' antivoff ' or ' anvitoff ' or ' caprilon ' or ' cl 65336 ' or ' cl65336 ' or ' cyclocapron ' or ' cyclokapron ' or ' cyklocapron ' or ' cyklokapron ' or ' exacyl ' or ' fibrinon ' or ' frenolyse ' or ' hemostan ' or ' hexacapron ' or ' hexakapron ' or ' kalnex ' or ' lysteda ' or ' micranex ' or ' para aminomethylcyclohexane carboxylic acid ' or ' rikaparin ' or ' ronex ' or ' theranex ' or ' tramic ' or ' tranex ' or ' tranexam ' or ' tranexanic acid ' or ' tranexic ' or ' trans 1 aminomethylcyclohexane 4 carboxylic acid ' or ' trans 4 aminomethyl cyclohexane 1 carboxylic acid ' or ' trans 4 aminomethyl cyclohexane carbonic acid ' or ' trans 4 aminomethyl cyclohexanecarboxylic acid ' or ' trans 4 aminomethylcyclohexane 1 carboxylic acid ' or ' trans 4 aminomethylcyclohexane carboxylic acid ' or ' trans 4 aminomethylcyclohexanecarboxylic acid ' or ' trans achma ' or ' trans amcha ' or ' trans aminomethyl cyclohexane carboxylic acid ' or ' trans aminomethylcyclohexane carboxylic acid ' or ' trans aminomethylcyclohexanecarboxylic acid ' or ' transamin ' or ' transaminomethylcyclohexane carboxylic acid ' or ' transexamic acid ' or ' traxamic ' or ' trenaxin ' or ' ugurol ').ab,hw,kf,ot,rn,sh,sy,ti,nm,mp. \| \| --- \| --- \| |
| 7 | \|  \| exp 'misoprostol'/ or (misoprostol or ' 11 ,16 dihydroxy 16 methyl 9 oxo 13 prostenoic acid methyl ester ' or ' 11,16 dihydroxy 16 methyl 9 oxoprost 13 enoic acid methyl ester ' or ' 15 deoxy 16 hydroxy 16 methylprostaglandin e1 methyl ester ' or ' 3 hydroxy 2 4 hydroxy 4 methyl 1 octenyl 5 oxocyclopentaneheptanoic acid methyl ester ' or ' angusta ' or ' cityl ' or ' cyprostol ' or ' cytolog ' or ' cytotec ' or ' gastotec ' or ' gastrul ' or ' gymiso ' or ' hemoprostol ' or ' isprelor ' or ' misel ' or ' misodel ' or ' misofar ' or ' misoone ' or ' misopress ' or ' misoprostil ' or ' misotrol ' or ' mispregnol ' or ' mysodelle ' or ' prostaglandin e1 methylester,15 deoxy 16 hydroxy 16 methyl ' or ' sc 29333 ' or ' sc 30249 ' or ' sc29333 ' or ' sc30249 ' or ' topogyne ' or ' u-miso ' or ' xp 16j ' or ' xp16j ').ab,hw,kf,ot,rn,sh,sy,ti,nm,mp. \| \| --- \| --- \| |
| 8 | \|  \| exp 'magnesium sulfate'/ or ('magnesium sulfate' or ' epsom salt ' or ' epsom salts ' or ' magnesium sulfate in dextrose ' or ' magnesium sulphate ' or 'mg longoral' or 'sulfamag' or 'sulmetin ' or 'sulmetine ').ab,hw,kf,ot,rn,sh,sy,ti,nm,mp. \| \| --- \| --- \| |
| 9 | exp 'penicillin G'/ or ('pencillin G' or ' angicillin ' or ' bacinol ' or ' benzopenicillin ' or ' benzoyl penicillin ' or ' benzyl penicillin ' or ' benzylpenicillin ' or ' benzylpenicillinic acid ' or ' benzylpenicilloyl ' or ' bupenna ' or ' cilligram ' or ' cilloral ' or ' cilopen ' or ' compocillin g ' or ' confets ' or ' conspen ' or ' depot penicillin ' or ' dis pen ' or ' dramcillin-100 ' or ' drop cillin ' or ' dropcillin ' or ' g penicillin ' or ' gelacillin ' or ' gynocillin ' or ' iricillin ' or ' jacillin ' or ' jenets ' or ' k cillin ' or ' keracillin ' or ' liquacillin ' or ' liquapen ' or ' megapen ' or ' nebutab ' or ' novocillin ' or ' novocilline ' or ' pen eff ' or ' penaire ' or ' penalev ' or ' penasoid ' or ' pendex ' or ' peneucin ' or ' penichrysin ' or ' penicillan ' or ' penicillin 9 ' or ' penicillin a ' or ' penicilline ' or ' penicilline g ' or ' penioral ' or ' penisem ' or ' pentab ' or ' penzal ' or ' perolety ' or ' pharmacillin ' or ' phenylmethylpenicillin ' or ' potassium g penicillin ' or ' pradupen ' or ' purapen g ' or ' readcillin ' or ' retarpen ' or ' semi synthetic penicillin ' or ' sol tabs ' or ' solupen ' or ' sopen ' or ' specillin g ' or ' stanpen 500 ' or ' sugracillin ' or ' til pen ' or ' unicilina ' or ' vagicillin ') .ab,hw,kf,ot,rn,sh,sy,ti,nm,mp. |
| 10 | \|  \| exp 'ampicillin'/ or (ampicillin or ' acillin ' or ' aldribid ' or ' aletmicina ' or ' alfasilin ' or ' alpha aminobenzylpenicillin ' or ' alphacin ' or ' ambiopi ' or ' amblocin ' or ' amblosin ' or ' amcill ' or ' amcillin ' or ' amficot ' or ' amfipen ' or ' aminobenzyl penicillin ' or ' aminobenzylpenicillin ' or ' amipenix ' or ' amoxi ' or ' amoxine ' or ' ampcillin ' or ' ampecu ' or ' ampen ' or ' ampenolet ' or ' ampensaar ' or ' ampexin ' or ' ampibex ' or ' ampiblan ' or ' ampicher ' or ' ampicil ' or ' ampicilin ' or ' ampicilina ' or ' ampiciline ' or ' ampicillin hydrate ' or ' ampicillin sensitivity ' or ' ampicillin sodium ' or ' ampicillin trihydrate ' or ' ampicillin ampicillin trihydrate ' or ' ampicilline ' or ' ampicin ' or ' ampicyn ' or ' ampidar ' or ' ampifen ' or ' ampiflex ' or ' ampiger ' or ' ampilag ' or ' ampilin ' or ' ampillin ' or ' ampimedin ' or ' ampipen ' or ' ampitenk ' or ' ampivral ' or ' ampkid ' or ' amplacilina ' or ' amplibin ' or ' ampliblan ' or ' amplital ' or ' amplivacil ' or ' ampolin ' or ' ampycin ' or ' amsapen ' or ' anglopen ' or ' anhydrous ampicillin ' or ' anhypen ' or ' apo-ampi ' or ' austrapen ' or ' ay 6108 ' or ' ay6108 ' or ' bayer 5427 ' or ' binotal ' or ' biocil ' or ' bremcillin ' or ' bridopen ' or ' britapen ' or ' britapen inj ' or ' brl 1341 ' or ' brl1341 ' or ' c 10575 ' or ' c10575 ' or ' camicil ' or ' cetampin ' or ' cimexillin ' or ' citicil ' or ' clovillin ' or ' copharcilin ' or ' dextro levo ampicillin ' or ' dhacillin ' or ' diferin ' or ' doktacillin ' or ' doltirol ' or ' domicillin ' or ' dotirol ' or ' duacillin ' or ' dumopen ' or ' eracillin ' or ' eurocin ' or ' excillin ' or ' extrapen ' or ' fontapen ' or ' gramcil ' or ' h-ambiotico ' or ' helvecillin ' or ' herpen ' or ' hi 63 ' or ' hi63 ' or ' hostes ' or ' ibimycin ' or ' ikacillin ' or ' intramed ' or ' iwacillin ' or ' jenampin ' or ' julphapen ' or ' ks r1 ' or ' marticil ' or ' mecil-n ' or ' neosensitabs ' or ' novo-ampicillin ' or ' nuvapen ' or ' omnipen ' or ' omnipen ampicillin ' or ' omnipen n ' or ' p 50 ' or ' pamecil ' or ' panacta ' or ' pen an ' or ' penbristol ' or ' penbritin ' or ' penbritin s ' or ' penicline ' or ' penodil ' or ' penstabil ' or ' pentrex ' or ' pentrexil ' or ' pentrexyl ' or ' pentrexyl k ' or ' petercillin ' or ' pfizerpen a ' or ' picylin ' or ' polycillin ' or ' polycillin n ' or ' polyflex ' or ' polypen ' or ' pricillin ' or ' primapen ' or ' princillin ' or ' principen ' or ' principen 125 ' or ' principen 250 ' or ' principen 500 ' or ' principen n ' or ' radiocillina ' or ' redicillin ' or ' rimacillin ' or ' roscillin ' or ' semicillin ' or ' servicillin ' or ' shacillin ' or ' sintelin ' or ' sodium ampicillin ' or ' standacillin ' or ' standcillin ' or ' synpenin ' or ' synthocilin ' or ' synthocillin ' or ' tolimal ' or ' totacillin ' or ' totacillin-n ' or ' totapen ' or ' trafarbiot ' or ' tricil ' or ' trifalicina ' or ' trihypen ' or ' trilaxin ' or ' ukapen ' or ' usampi ' or ' vacillin ' or ' viccillin ' or ' vidopen ' or ' virucil ' or ' vitapen ').ab,hw,kf,ot,rn,sh,sy,ti,nm,mp. \| \| --- \| --- \| |
| 11 | exp 'gentamicins'/ or (' gentamicin' or ' adelanin ' or ' alcomicin ' or ' apigent ' or ' apogen ' or ' apoten ' or ' azupel ' or ' bactiderm ' or ' biogaracin ' or ' bristagen ' or ' cidomycin ' or ' danigen ' or ' dermogen ' or ' dianfarma ' or ' dispagent ' or ' duragentam ' or ' duragentamicin ' or ' epigent ' or ' frieso gent ' or ' g-mycin ' or ' garabiotic ' or ' garalone ' or ' garamicin ' or ' garamicina ' or ' garamicina cream ' or ' garamicina crema ' or ' garamicina oftalmica ' or ' garamycin ' or ' garamycin ophthalmic ' or ' garamycin topical ' or ' garbilocin ' or ' gencin ' or ' gendril ' or ' genoptic ' or ' genoptic liquifilm ' or ' genoptic sop ' or ' genrex ' or ' gensumycin ' or ' genta 20 ' or ' genta 50 ' or ' genta grin ' or ' genta-gobens ' or ' gentabiotic ' or ' gentabiox ' or ' gentac ' or ' gentacidin ' or ' gentacin ' or ' gentacor ' or ' gentacyl ' or ' gentafair ' or ' gentagram ' or ' gentak ' or ' gental ' or ' gentaline ' or ' gentalline ' or ' gentalol ' or ' gentalyn ' or ' gentalyn oftalmico-otico ' or ' gentamax ' or ' gentame ' or ' gentamedical ' or ' gentamen ' or ' gentamerck ' or ' gentamicin sulfate ' or ' gentamicin sulfate in sodium chloride ' or ' gentamicin sulphate ' or ' gentamicin sulphate in sodium chloride ' or ' gentamicins ' or ' gentamina ' or ' gentamycin ' or ' gentamycin a1 ' or ' gentamycin cream ' or ' gentamycin sulfate ' or ' gentamycin sulphate ' or ' gentamycine ' or ' gentamyl ' or ' gentamytrex ' or ' gentamytrex ophthiole ' or ' gentaplus ' or ' gentarad ' or ' gentasil ' or ' gentasol ' or ' gentasone ' or ' gentasporin ' or ' gentatrim ' or ' genticin ' or ' genticina ' or ' genticyn ' or ' genticyn hg ' or ' gentiderm ' or ' gentimycin ' or ' gentocin ' or ' gentogram ' or ' gentomycin ' or ' genum ' or ' geomycine ' or ' gevramycin ' or ' grammicin ' or ' hexamycin ' or ' isotonic gentamicin sulfate ' or ' isotonic gentamicin sulphate ' or ' jenamicin ' or ' konigen ' or ' lacromycin ' or ' lisagent ' or ' martigenta ' or ' megental ' or ' migenta ' or ' miragenta ' or ' miramycin ' or ' nichogencin ' or ' nsc 82261 ' or ' nsc82261 ' or ' obogen ' or ' ocu-mycin ' or ' ocugenta ' or ' oftagen ' or ' ophtagram ' or ' opthagen ' or ' opti-genta ' or ' optigen ' or ' ottogenta ' or ' pyogenta ' or ' refobacin ' or ' refobacin palacos r ' or ' ribomicin ' or ' rigaminol ' or ' rocy gen ' or ' rovixida ' or ' rupegen ' or ' sagestam eye drops ' or ' sch 9724 ' or ' sch9724 ' or ' sedanazin ' or ' servigenta ' or ' skinfect ' or ' sulmycin ' or ' tangyn ' or ' terramycin n augensalbe ' or ' terramycin n augentropfen ' or ' u-gencin ' or ' versigen ' or ' yectamicina ').ab,hw,kf,ot,rn,sh,sy,ti,nm,mp. |
| 12 | exp 'cefazolin'/ or (((' ancef ' or ' ancef in dextrose 5 ' or ' ancef in sodium chloride ' or ' ansef ' or ' anzolin ' or ' basocef ' or ' biozolin ' or ' cefacidal ' or ' cefamezin ' or ' cefamezine ' or ' cefarad ' or ' cefazin ' or ' cefazol ' or ' cefazolin) and dextrose ') or ' cefazolin sodium ' or ' cefazolina ' or ' cefazoline ' or ' cefazoline panpharma ' or ' celmetin ' or ' cephamezin ' or ' cephazolin ' or ' cezolin ' or ' elzogram ' or ' faxilen ' or ' fazolin ' or ' fonvicol ' or ' gramaxin ' or ' intrazolina ' or ' izacef ' or ' kefarin ' or ' kefazin ' or ' kefazolin ' or ' kefzol ' or ' kezolin ' or ' kofatol ' or ' kurgan ' or ' lupex ' or ' oricef ' or ' orizolin ' or ' reflin ' or ' sanzol ' or ' stancef ' or ' stazolin ' or ' surzolin ' or ' totacef ' or ' uzolin ' or ' vifazolin ' or ' vulmizolin ' or ' zaconil ' or ' zolecef ' or ' zolicef ' or ' zolidina ') .ab,hw,kf,ot,rn,sh,sy,ti,nm,mp. |
| 13 | \|  \| exp 'metronidazole'/ or (metronidazole ti,ab or ' acea gel ' or ' acromona ' or ' amevan ' or ' amiyodazol ' or ' anaerobex ' or ' anerobia ' or ' apo-metronidazole ' or ' arcazol ' or ' arilin ' or ' ariline ' or ' aristogyl ' or ' asiazole ' or ' asuzol ' or ' atrivyl ' or ' bayer 5360 ' or ' biotazol ' or ' camezol ' or ' clont ' or ' cont ' or ' danizol ' or ' deflamon ' or ' dumozol ' or ' elyzol ' or ' endazole ' or ' entizol ' or ' farnat ' or ' fladex ' or ' flagenase ' or ' flagesol ' or ' flagil ' or ' flagizole ' or ' flagyl ' or ' flagyl 375 ' or ' flagyl compak ' or ' flagyl er ' or ' flagyl i v ' or ' flagyl i v rtu ' or ' flasinyl ' or ' flazol ' or ' flegyl ' or ' fossyol ' or ' frotin ' or ' giardyl ' or ' gineflavir ' or ' helminzol ' or ' ivemetro ' or ' klion ' or ' kreucosan ' or ' marphazole ' or ' metragyl ' or ' metranidazole ' or ' metric 21 ' or ' metro i v ' or ' metrocream ' or ' metrodinazole ' or ' metrogel ' or ' metrogel vaginal ' or ' metrogyl ' or ' metrogyl unique ' or ' metrolag ' or ' metrolex ' or ' metrolotion ' or ' metromidol ' or ' metronid ' or ' metronidanol ' or ' metronidasol ' or ' metronidazol ' or ' metronidazol mckesson ' or ' metronidazole hydrochloride ' or ' metronidazole injection ' or ' metronidazole iv ' or ' metronidazone ' or ' metronide ' or ' metronil ' or ' metronizadole ' or ' metrozin ' or ' metrozine ' or ' metryl ' or ' monasin ' or ' nalox ' or ' nidazol ' or ' nor-metrogel ' or ' noritate ' or ' noritate cream ' or ' novonidazole ' or ' nsc 50364 ' or ' orvagil ' or ' otrozol ' or ' patryl ' or ' protogyl ' or ' protostat ' or ' protostate ' or ' protozol ' or ' qualigyl ' or ' rathimed n ' or ' robaz ' or ' rodazid ' or ' rosaced gel ' or ' rosalox ' or ' rozacreme ' or ' rozagel ' or ' rozex ' or ' rozex gel ' or ' rp 8823 ' or ' satric ' or ' sc 32642 ' or ' servizol ' or ' sharizole ' or ' supplin ' or ' surimol ' or ' takimetol ' or ' torgyl ' or ' trichazol ' or ' trichex ' or ' tricho cordes ' or ' trichogynaedron ' or ' trichomol ' or ' trichopol ' or ' trichopole ' or ' trichozole ' or ' tricocet ' or ' tricom ' or ' triconex ' or ' tricowas b ' or ' trikacide ' or ' trikozol ' or ' trivazol ' or ' trogiar ' or ' unigo ' or ' vagimid ' or ' vandazole ' or ' zadstat ' or ' zidoval gel ').ab,hw,kf,ot,rn,sh,sy,ti,nm,mp. \| \| --- \| --- \| |
| 14 | 1 or 2 or 3 or 4 or 5 or 6 or 7 or 8 or 9 or 10 or 11 or 12 or 13 |
| 15 | \|  \| exp 'drug contamination'/ or exp 'counterfeit drug'/ or 'drug contamination'.ti,ab. or counterfeit.ti,ab. or fake.ti,ab. or impurity.ti,ab. or impurities.ti,ab. or exp 'drug quality'/ or exp 'drug monitoring'/ or 'drug monitoring'.ti,ab. OR exp 'Substandard Drugs'/ \| \| --- \| --- \| |
| 16 | exp 'drug storage'/ OR exp 'drug stability'/ OR exp 'humidity'/ OR exp ' Medication Errors’/ OR 'Medication errors'.ti,ab. OR 'drug stablilty'.ti,ab. OR storage.ti,ab. OR potency.ti,ab. OR potencies.ti,ab OR 'active pharmacological ingredient'.ti,ab OR 'active pharmacological ingredients'.ti,ab. OR 'active pharmacological ingredient'.ti,ab. OR 'non active'.ti,ab. OR (light ADJ2 expos*).ti,ab OR stable.ti,ab OR potency.ti,ab. OR potencies.ti,ab. |
| 17 | exp Quality of Health Care / or standard.fs. or exp 'quality control'/ or 'quality control'.ti,ab. OR substandard.ti,ab. or 'non-compliant'.ti,ab. or 'non-complianance'.ti,ab. or inadequate.ti,ab. or surveillance.ti,ab. or specification*.ti,ab. or 'failure rate'.ti,ab. or 'failure rates'.ti,ab. or spurious.ti,ab. or degradat*.ti,ab. or unregistered.ti,ab. or insufficien*.ti,ab. |
| 18 | \|  \| 14 and 15 \| \| --- \| --- \| |
| 19 | 14 and 16 and 17 |
| 20 | \|  \| 18 or 19 \| \| --- \| --- \| |

|  | LILACS (via Bireme) |
| --- | --- |
| 1 | (oxytocin OR carbetocin OR ergometrine OR (tranexamic acid) OR misoprostol OR (magnesium sulfate) OR (penicillin g) OR ampicillin OR gentamicin OR cefazolin OR metronidazole ) AND (quality OR fake OR ((heat OR light) AND exposure) OR standard OR failure OR counterfeit OR substandard OR (drug AND storage) OR contamination OR impurity OR potency OR impurities OR (drug monitoring) OR (drug surveillance) OR (active pharmacological)) |

| # | Medline (via Ovid) |
| --- | --- |
| 1 | exp 'uterotonic agent'/ or ('uterotonic agent' or 'uterotonic agents').ab,du,dy,kw,mf,ot,ox,rn,sh,ti,tn,dq. |
| 2 | exp oxytocin/ or ('orasthin' or ' orastina ' or ' oxitocin ' or ' oxiton inj ' or ' oxitone ' or ' oxystin ' or ' oxytan ' or ' oxytocina ' or ' oxytocine ' or ' pareton ' or ' partacon ' or ' partocon ' or ' partolact ' or ' partoxin ' or ' physormon ' or ' pitocin ' or ' piton ' or ' pituilobine ' or ' pitupartin ' or ' solvoxine ' or ' synpitan ' or ' syntocinon ' or ' tranoxy ' or ' utedrin ' or ' uteracon ' or ' uterason ' or ' utron ' or ' xitocin ').ab,du,dy,kw,mf,ot,ox,rn,sh,ti,tn,dq. |
| 3 | exp carbetocin/ or (carbetocina' or crinesal' or duratobal' or duratocin' or pabal').ab,du,dy,kw,mf,ot,ox,rn,sh,ti,tn,dq. |
| 4 | exp 8beta ergometrine/ or (8beta ergometrine' or 'd lysergic acid 1 ydroxymethylethylamide' or 'd lysergic acid l 2 propanolamide' or 'ergobasine' or 'ergoklinine' or 'ergometrin' or 'ergonovine' or 'ergostetrine' or ergotocine' or 'ergotrate' or 'hydroxypropyllysergamide' or 'lysergic acid propanolamide').ab,du,dy,kw,mf,ot,ox,rn,sh,ti,tn,dq. |
| 5 | exp 'carbetocin'/ or (carbetocina' or crinesal' or duratobal' or duratocin' or pabal').ab,du,dy,kw,mf,ot,ox,rn,sh,ti,tn,dq. |
| 6 | exp 'tranexamic acid'/ or (' aminomethyl cyclohexanecarboxylic ' or ' 4 aminomethylcyclohexanecarbonic acid ' or ' 4 aminomethylcyclohexanecarboxylic acid ' or ' amca ' or ' AMCHA ' or ' amchafibrin ' or ' amikapron ' or ' amino methylcyclohexane* ' or ' aminomethyl cyclohexane ' or ' aminomethyl cyclohexane* ' or ' minomethylcyclohexanecarboxylic* ' or ' amstat ' or ' anexan ' or ' antivoff ' or ' anvitoff ' or ' caprilon ' or ' cl 65336 ' or ' cl65336 ' or ' cyclocapron ' or ' cyclokapron ' or ' cyklocapron ' or ' cyklokapron ' or ' exacyl ' or ' fibrinon ' or ' frenolyse ' or ' hemostan ' or ' hexacapron ' or ' hexakapron ' or ' kalnex ' or ' lysteda ' or ' micranex ' or ' para aminomethylcyclohexane carboxylic acid ' or ' rikaparin ' or ' ronex ' or ' theranex ' or ' tramic ' or ' tranex ' or ' tranexam ' or ' tranexanic acid ' or ' tranexic ' or ' trans 1 aminomethylcyclohexane 4 carboxylic acid ' or ' trans 4 aminomethyl cyclohexane 1 carboxylic acid ' or ' trans 4 aminomethyl cyclohexane carbonic acid ' or ' trans 4 aminomethyl cyclohexanecarboxylic acid ' or ' trans 4 aminomethylcyclohexane 1 carboxylic acid ' or ' trans 4 aminomethylcyclohexane carboxylic acid ' or ' trans 4 aminomethylcyclohexanecarboxylic acid ' or ' trans achma ' or ' trans amcha ' or ' trans aminomethyl cyclohexane carboxylic acid ' or ' trans aminomethylcyclohexane carboxylic acid ' or ' trans aminomethylcyclohexanecarboxylic acid ' or ' transamin ' or ' transaminomethylcyclohexane carboxylic acid ' or ' transexamic acid ' or ' traxamic ' or ' trenaxin ' or ' ugurol ').ab,du,dy,kw,mf,ot,ox,rn,sh,ti,tn,dq. |
| 7 | exp 'misoprostol'/ or (misoprostol or ' 11 ,16 dihydroxy 16 methyl 9 oxo 13 prostenoic acid methyl ester ' or ' 11,16 dihydroxy 16 methyl 9 oxoprost 13 enoic acid methyl ester ' or ' 15 deoxy 16 hydroxy 16 methylprostaglandin e1 methyl ester ' or ' 3 hydroxy 2 4 hydroxy 4 methyl 1 octenyl 5 oxocyclopentaneheptanoic acid methyl ester ' or ' angusta ' or ' cityl ' or ' cyprostol ' or ' cytolog ' or ' cytotec ' or ' gastotec ' or ' gastrul ' or ' gymiso ' or ' hemoprostol ' or ' isprelor ' or ' misel ' or ' misodel ' or ' misofar ' or ' misoone ' or ' misopress ' or ' misoprostil ' or ' misotrol ' or ' mispregnol ' or ' mysodelle ' or ' prostaglandin e1 methylester,15 deoxy 16 hydroxy 16 methyl ' or ' sc 29333 ' or ' sc 30249 ' or ' sc29333 ' or ' sc30249 ' or ' topogyne ' or ' u-miso ' or ' xp 16j ' or ' xp16j ').ab,du,dy,kw,mf,ot,ox,rn,sh,ti,tn,dq. |
| 8 | exp 'magnesium sulfate'/ or ('magnesium sulfate' or ' epsom salt ' or ' epsom salts ' or ' magnesium sulfate in dextrose ' or ' magnesium sulphate ' or 'mg longoral' or 'sulfamag' or 'sulmetin ' or 'sulmetine ').ab,du,dy,kw,mf,ot,ox,rn,sh,ti,tn,dq. |
| 9 | exp 'penicillin G'/ or ('pencillin G' or ' angicillin ' or ' bacinol ' or ' benzopenicillin ' or ' benzoyl penicillin ' or ' benzyl penicillin ' or ' benzylpenicillin ' or ' benzylpenicillinic acid ' or ' benzylpenicilloyl ' or ' bupenna ' or ' cilligram ' or ' cilloral ' or ' cilopen ' or ' compocillin g ' or ' confets ' or ' conspen ' or ' depot penicillin ' or ' dis pen ' or ' dramcillin-100 ' or ' drop cillin ' or ' dropcillin ' or ' g penicillin ' or ' gelacillin ' or ' gynocillin ' or ' iricillin ' or ' jacillin ' or ' jenets ' or ' k cillin ' or ' keracillin ' or ' liquacillin ' or ' liquapen ' or ' megapen ' or ' nebutab ' or ' novocillin ' or ' novocilline ' or ' pen eff ' or ' penaire ' or ' penalev ' or ' penasoid ' or ' pendex ' or ' peneucin ' or ' penichrysin ' or ' penicillan ' or ' penicillin 9 ' or ' penicillin a ' or ' penicilline ' or ' penicilline g ' or ' penioral ' or ' penisem ' or ' pentab ' or ' penzal ' or ' perolety ' or ' pharmacillin ' or ' phenylmethylpenicillin ' or ' potassium g penicillin ' or ' pradupen ' or ' purapen g ' or ' readcillin ' or ' retarpen ' or ' semi synthetic penicillin ' or ' sol tabs ' or ' solupen ' or ' sopen ' or ' specillin g ' or ' stanpen 500 ' or ' sugracillin ' or ' til pen ' or ' unicilina ' or ' vagicillin ').ab,du,dy,kw,mf,ot,ox,rn,sh,ti,tn,dq. |
| 10 | exp 'ampicillin'/ or (ampicillin or ' acillin ' or ' aldribid ' or ' aletmicina ' or ' alfasilin ' or ' alpha aminobenzylpenicillin ' or ' alphacin ' or ' ambiopi ' or ' amblocin ' or ' amblosin ' or ' amcill ' or ' amcillin ' or ' amficot ' or ' amfipen ' or ' aminobenzyl penicillin ' or ' aminobenzylpenicillin ' or ' amipenix ' or ' amoxi ' or ' amoxine ' or ' ampcillin ' or ' ampecu ' or ' ampen ' or ' ampenolet ' or ' ampensaar ' or ' ampexin ' or ' ampibex ' or ' ampiblan ' or ' ampicher ' or ' ampicil ' or ' ampicilin ' or ' ampicilina ' or ' ampiciline ' or ' ampicillin hydrate ' or ' ampicillin sensitivity ' or ' ampicillin sodium ' or ' ampicillin trihydrate ' or ' ampicillin ampicillin trihydrate ' or ' ampicilline ' or ' ampicin ' or ' ampicyn ' or ' ampidar ' or ' ampifen ' or ' ampiflex ' or ' ampiger ' or ' ampilag ' or ' ampilin ' or ' ampillin ' or ' ampimedin ' or ' ampipen ' or ' ampitenk ' or ' ampivral ' or ' ampkid ' or ' amplacilina ' or ' amplibin ' or ' ampliblan ' or ' amplital ' or ' amplivacil ' or ' ampolin ' or ' ampycin ' or ' amsapen ' or ' anglopen ' or ' anhydrous ampicillin ' or ' anhypen ' or ' apo-ampi ' or ' austrapen ' or ' ay 6108 ' or ' ay6108 ' or ' bayer 5427 ' or ' binotal ' or ' biocil ' or ' bremcillin ' or ' bridopen ' or ' britapen ' or ' britapen inj ' or ' brl 1341 ' or ' brl1341 ' or ' c 10575 ' or ' c10575 ' or ' camicil ' or ' cetampin ' or ' cimexillin ' or ' citicil ' or ' clovillin ' or ' copharcilin ' or ' dextro levo ampicillin ' or ' dhacillin ' or ' diferin ' or ' doktacillin ' or ' doltirol ' or ' domicillin ' or ' dotirol ' or ' duacillin ' or ' dumopen ' or ' eracillin ' or ' eurocin ' or ' excillin ' or ' extrapen ' or ' fontapen ' or ' gramcil ' or ' h-ambiotico ' or ' helvecillin ' or ' herpen ' or ' hi 63 ' or ' hi63 ' or ' hostes ' or ' ibimycin ' or ' ikacillin ' or ' intramed ' or ' iwacillin ' or ' jenampin ' or ' julphapen ' or ' ks r1 ' or ' marticil ' or ' mecil-n ' or ' neosensitabs ' or ' novo-ampicillin ' or ' nuvapen ' or ' omnipen ' or ' omnipen ampicillin ' or ' omnipen n ' or ' p 50 ' or ' pamecil ' or ' panacta ' or ' pen an ' or ' penbristol ' or ' penbritin ' or ' penbritin s ' or ' penicline ' or ' penodil ' or ' penstabil ' or ' pentrex ' or ' pentrexil ' or ' pentrexyl ' or ' pentrexyl k ' or ' petercillin ' or ' pfizerpen a ' or ' picylin ' or ' polycillin ' or ' polycillin n ' or ' polyflex ' or ' polypen ' or ' pricillin ' or ' primapen ' or ' princillin ' or ' principen ' or ' principen 125 ' or ' principen 250 ' or ' principen 500 ' or ' principen n ' or ' radiocillina ' or ' redicillin ' or ' rimacillin ' or ' roscillin ' or ' semicillin ' or ' servicillin ' or ' shacillin ' or ' sintelin ' or ' sodium ampicillin ' or ' standacillin ' or ' standcillin ' or ' synpenin ' or ' synthocilin ' or ' synthocillin ' or ' tolimal ' or ' totacillin ' or ' totacillin-n ' or ' totapen ' or ' trafarbiot ' or ' tricil ' or ' trifalicina ' or ' trihypen ' or ' trilaxin ' or ' ukapen ' or ' usampi ' or ' vacillin ' or ' viccillin ' or ' vidopen ' or ' virucil ' or ' vitapen ').ab,du,dy,kw,mf,ot,ox,rn,sh,ti,tn,dq. |
| 11 | exp 'gentamicin'/ or (' gentamicin' or ' adelanin ' or ' alcomicin ' or ' apigent ' or ' apogen ' or ' apoten ' or ' azupel ' or ' bactiderm ' or ' biogaracin ' or ' bristagen ' or ' cidomycin ' or ' danigen ' or ' dermogen ' or ' dianfarma ' or ' dispagent ' or ' duragentam ' or ' duragentamicin ' or ' epigent ' or ' frieso gent ' or ' g-mycin ' or ' garabiotic ' or ' garalone ' or ' garamicin ' or ' garamicina ' or ' garamicina cream ' or ' garamicina crema ' or ' garamicina oftalmica ' or ' garamycin ' or ' garamycin ophthalmic ' or ' garamycin topical ' or ' garbilocin ' or ' gencin ' or ' gendril ' or ' genoptic ' or ' genoptic liquifilm ' or ' genoptic sop ' or ' genrex ' or ' gensumycin ' or ' genta 20 ' or ' genta 50 ' or ' genta grin ' or ' genta-gobens ' or ' gentabiotic ' or ' gentabiox ' or ' gentac ' or ' gentacidin ' or ' gentacin ' or ' gentacor ' or ' gentacyl ' or ' gentafair ' or ' gentagram ' or ' gentak ' or ' gental ' or ' gentaline ' or ' gentalline ' or ' gentalol ' or ' gentalyn ' or ' gentalyn oftalmico-otico ' or ' gentamax ' or ' gentame ' or ' gentamedical ' or ' gentamen ' or ' gentamerck ' or ' gentamicin sulfate ' or ' gentamicin sulfate in sodium chloride ' or ' gentamicin sulphate ' or ' gentamicin sulphate in sodium chloride ' or ' gentamicins ' or ' gentamina ' or ' gentamycin ' or ' gentamycin a1 ' or ' gentamycin cream ' or ' gentamycin sulfate ' or ' gentamycin sulphate ' or ' gentamycine ' or ' gentamyl ' or ' gentamytrex ' or ' gentamytrex ophthiole ' or ' gentaplus ' or ' gentarad ' or ' gentasil ' or ' gentasol ' or ' gentasone ' or ' gentasporin ' or ' gentatrim ' or ' genticin ' or ' genticina ' or ' genticyn ' or ' genticyn hg ' or ' gentiderm ' or ' gentimycin ' or ' gentocin ' or ' gentogram ' or ' gentomycin ' or ' genum ' or ' geomycine ' or ' gevramycin ' or ' grammicin ' or ' hexamycin ' or ' isotonic gentamicin sulfate ' or ' isotonic gentamicin sulphate ' or ' jenamicin ' or ' konigen ' or ' lacromycin ' or ' lisagent ' or ' martigenta ' or ' megental ' or ' migenta ' or ' miragenta ' or ' miramycin ' or ' nichogencin ' or ' nsc 82261 ' or ' nsc82261 ' or ' obogen ' or ' ocu-mycin ' or ' ocugenta ' or ' oftagen ' or ' ophtagram ' or ' opthagen ' or ' opti-genta ' or ' optigen ' or ' ottogenta ' or ' pyogenta ' or ' refobacin ' or ' refobacin palacos r ' or ' ribomicin ' or ' rigaminol ' or ' rocy gen ' or ' rovixida ' or ' rupegen ' or ' sagestam eye drops ' or ' sch 9724 ' or ' sch9724 ' or ' sedanazin ' or ' servigenta ' or ' skinfect ' or ' sulmycin ' or ' tangyn ' or ' terramycin n augensalbe ' or ' terramycin n augentropfen ' or ' u-gencin ' or ' versigen ' or ' yectamicina ').ab,du,dy,kw,mf,ot,ox,rn,sh,ti,tn,dq. |
| 12 | exp 'cefazolin'/ or (((' ancef ' or ' ancef in dextrose 5 ' or ' ancef in sodium chloride ' or ' ansef ' or ' anzolin ' or ' basocef ' or ' biozolin ' or ' cefacidal ' or ' cefamezin ' or ' cefamezine ' or ' cefarad ' or ' cefazin ' or ' cefazol ' or ' cefazolin) and dextrose ') or ' cefazolin sodium ' or ' cefazolina ' or ' cefazoline ' or ' cefazoline panpharma ' or ' celmetin ' or ' cephamezin ' or ' cephazolin ' or ' cezolin ' or ' elzogram ' or ' faxilen ' or ' fazolin ' or ' fonvicol ' or ' gramaxin ' or ' intrazolina ' or ' izacef ' or ' kefarin ' or ' kefazin ' or ' kefazolin ' or ' kefzol ' or ' kezolin ' or ' kofatol ' or ' kurgan ' or ' lupex ' or ' oricef ' or ' orizolin ' or ' reflin ' or ' sanzol ' or ' stancef ' or ' stazolin ' or ' surzolin ' or ' totacef ' or ' uzolin ' or ' vifazolin ' or ' vulmizolin ' or ' zaconil ' or ' zolecef ' or ' zolicef ' or ' zolidina ').ab,du,dy,kw,mf,ot,ox,rn,sh,ti,tn,dq. |
| 13 | exp 'metronidazole'/ or (metronidazole ti,ab or ' acea gel ' or ' acromona ' or ' amevan ' or ' amiyodazol ' or ' anaerobex ' or ' anerobia ' or ' apo-metronidazole ' or ' arcazol ' or ' arilin ' or ' ariline ' or ' aristogyl ' or ' asiazole ' or ' asuzol ' or ' atrivyl ' or ' bayer 5360 ' or ' biotazol ' or ' camezol ' or ' clont ' or ' cont ' or ' danizol ' or ' deflamon ' or ' dumozol ' or ' elyzol ' or ' endazole ' or ' entizol ' or ' farnat ' or ' fladex ' or ' flagenase ' or ' flagesol ' or ' flagil ' or ' flagizole ' or ' flagyl ' or ' flagyl 375 ' or ' flagyl compak ' or ' flagyl er ' or ' flagyl i v ' or ' flagyl i v rtu ' or ' flasinyl ' or ' flazol ' or ' flegyl ' or ' fossyol ' or ' frotin ' or ' giardyl ' or ' gineflavir ' or ' helminzol ' or ' ivemetro ' or ' klion ' or ' kreucosan ' or ' marphazole ' or ' metragyl ' or ' metranidazole ' or ' metric 21 ' or ' metro i v ' or ' metrocream ' or ' metrodinazole ' or ' metrogel ' or ' metrogel vaginal ' or ' metrogyl ' or ' metrogyl unique ' or ' metrolag ' or ' metrolex ' or ' metrolotion ' or ' metromidol ' or ' metronid ' or ' metronidanol ' or ' metronidasol ' or ' metronidazol ' or ' metronidazol mckesson ' or ' metronidazole hydrochloride ' or ' metronidazole injection ' or ' metronidazole iv ' or ' metronidazone ' or ' metronide ' or ' metronil ' or ' metronizadole ' or ' metrozin ' or ' metrozine ' or ' metryl ' or ' monasin ' or ' nalox ' or ' nidazol ' or ' nor-metrogel ' or ' noritate ' or ' noritate cream ' or ' novonidazole ' or ' nsc 50364 ' or ' orvagil ' or ' otrozol ' or ' patryl ' or ' protogyl ' or ' protostat ' or ' protostate ' or ' protozol ' or ' qualigyl ' or ' rathimed n ' or ' robaz ' or ' rodazid ' or ' rosaced gel ' or ' rosalox ' or ' rozacreme ' or ' rozagel ' or ' rozex ' or ' rozex gel ' or ' rp 8823 ' or ' satric ' or ' sc 32642 ' or ' servizol ' or ' sharizole ' or ' supplin ' or ' surimol ' or ' takimetol ' or ' torgyl ' or ' trichazol ' or ' trichex ' or ' tricho cordes ' or ' trichogynaedron ' or ' trichomol ' or ' trichopol ' or ' trichopole ' or ' trichozole ' or ' tricocet ' or ' tricom ' or ' triconex ' or ' tricowas b ' or ' trikacide ' or ' trikozol ' or ' trivazol ' or ' trogiar ' or ' unigo ' or ' vagimid ' or ' vandazole ' or ' zadstat ' or ' zidoval gel ').ab,du,dy,kw,mf,ot,ox,rn,sh,ti,tn,dq. |
| 14 | 1 or 2 or 3 or 4 or 5 or 6 or 7 or 8 or 9 or 10 or 11 or 12 or 13 |
| 15 | exp 'drug contamination'/ or exp 'counterfeit drug'/ or 'drug contamination'.ti,ab. or counterfeit.ti,ab. or fake.ti,ab. or impurity.ti,ab. or impurities.ti,ab. or exp 'drug quality'/ or exp 'drug monitoring'/ or 'drug monitoring'.ti,ab. |
| 16 | exp 'drug storage'/ or exp 'drug stability'/ or exp 'humidity'/ or exp ' heat sensitivity'/ or 'Medication errors'.ti,ab. or 'drug stablilty'.ti,ab. or storage.ti,ab. or potency.ti,ab. or potencies.ti,ab. or 'active pharmacological ingredient'.ti,ab. or 'active pharmacological ingredients'.ti,ab. or 'active pharmacological ingredient'.ti,ab. or 'non active'.ti,ab. or (light adj2 expos*).ti,ab. or stable.ti,ab. or potency.ti,ab. or potencies.ti,ab. |
| 17 | exp health care quality/ or exp 'standard'/ or exp 'quality control'/ or 'quality control'.ti,ab. or substandard.ti,ab. or 'non-compliant'.ti,ab. or 'non-complianance'.ti,ab. or inadequate.ti,ab. or surveillance.ti,ab. or specification*.ti,ab. or 'failure rate'.ti,ab. or 'failure rates'.ti,ab. or spurious.ti,ab. or degradat*.ti,ab. or unregistered.ti,ab. or insufficien*.ti,ab. |
| 18 | 14 and 15 |
| 19 | 14 and 16 and 17 |
| 20 | 18 or 19 |
