# Supplementary material for: Quality of medicines for life-threatening pregnancy complications in low- and middle-income countries: A systematic review
Source: PLoS One. 2020 Jul 10;15(7):e0236060. doi: 10.1371/journal.pone.0236060 (PMC7351160; doi:10.1371/journal.pone.0236060)
Supplement: S2 Appendix — (DOCX) [file pone.0236060.s002.docx]

**S2 Appendix. List of 200 excluded studies and reasons**

Reasons for exclusion of study or report:

| Code | Reason | N studies |
| --- | --- | --- |
| A | Not a 1ary study with data on quality of medicine | 67 |
| B | Did not assess one of the pre-selected medicines in our list | 47 |
| C | Antibiotic in non-parenteral route | 37 |
| D | Simulation study to test stability/other properties | 18 |
| E | Abstract or letter on a study but insufficient information | 10 |
| F | Medicine only in high income country | 11 |
| G | Laboratory methods to assess medicines | 6 |
| H | Letter or editorial | 3 |
| I | Unable to get full text | 1 |

| **Reference** | **Resaon** |
| --- | --- |
| Abu-Reid IO, El-Samani SA, Hag Omer AI, Khalil NY, Ma hgoub KM, Everitt G, et al. Stability of drugs in the tropics. A study in Sudan. International Pharmacy Journal. 1990;4(1):6-10. | D |
| Acarturk F. Evaluation of the stability of ampicillin tablets. Gazi Universitesi Eczacilik Fakultesi Dergisi. 1988;5(2):139-46. | D |
| Afu S. Incidence of substandard drug in developing countries [2]. Tropical Medicine and International Health. 1999;4(1):73. | H |
| Ahmad M, Rehman Khan A. Quality control of pharmaceuticals by high-performance liquid chromatography. Journal of the Pakistan Medical Association. 1984;34(2):35-8. | A |
| Ajala TO, Oreagba MI, Odeku OA. The pharmaceutical equivalence and stability of multisource metronidazole suspensions. African journal of medicine and medical sciences. 2014;43(2):139-46. | C |
| Akunyili DN, Nnani IPC. Risk of medicines: Counterfeit drugs. International Journal of Risk and Safety in Medicine. 2004;16(3):181-90. | A |
| Aleanizy FS, Al-Eid H, El Tahir E, Alqahtani F, Al-Gohary O. Stability and in vitro dissolution studies of metronidazole tablets and infusions. Dissolution Technologies. 2017;24(2):22-7. | D |
| Allen Jr LV, Erickson IMA. Stability of ketoconazole, metolazone, metronidazole, procainamide hydrochloride, and spironolactone in extemporaneously compounded oral liquids. American Journal of Health-System Pharmacy. 1996;53(17):2073-8. | C |
| Aminu N, Gwarzo MS. The eminent threats of counterfeit drugs to quality health care delivery in africa: Updates on consequences and way forward. Asian Journal of Pharmaceutical and Clinical Research. 2017;10(7):82-6. | A |
| Andriollo O, Machuron L, Videau JY, Abelli C, Plot S, Muller D. Supplies for humanitarian aid and development countries: The quality of essential multisource drugs. STP Pharma Pratiques. 1998;8(2):137-55. | A |
| Anisfeld MH. Counterfeit pharmaceuticals and the International Pharmaceutical Federation (FIP) working group on counterfeit medicines. Journal of Pharmacy Practice. 2006;19(3):178-81. | A |
| Antimicrobial resistance: What does medicine quality have to do with it? (report, available on line) Antimicrobial Resistance and Medicine Quality. Pisani for AMR Review, November 2015 http://apps.who.int/medicinedocs/documents/s22186en/s22186en.pdf | A |
| Appiah B. US Pharmacopeia fighting counterfeit medicines in Africa. CMAJ. 2013 Oct 1;185(14):E666. doi: 10.1503/cmaj.109-4571 | A |
| Arya SC. Global climate warming and performance of therapeutic agents in obstetrics and gynecology. International Journal of Gynecology and Obstetrics. 1999;65(2):209-10. | A |
| Arya SC. Global warming and performance of antibiotics and antibacterial agents. International Pharmacy Journal. 1999;13(2):41-2. | H |
| Arya SC. Global warming and the performance of drugs used to treat parasitic and other diseases. Annals of Tropical Medicine and Parasitology. 1999;93(2):207-8. | A |
| Arya SC. Potency and bioavailability of therapeutic formulations during their field usages. International Journal of Pharmaceutics. 1999;184(2):263-4. | H |
| Ashenafi D, Van Hemelrijck E, Chopra S, Hoogmartens J, Adams E. Liquid chromatographic analysis of oxytocin and its related substances. Journal of Pharmaceutical and Biomedical Analysis. 2010;51(1):24-9. | G |
| Avianto, P.; Mahfudz,; Suharjono,; Isnaeni,; Alderman, C. P. In vitro equivalence of generic and branded amoxicillin tablet by microbiological assay method Journal of Basic and Clinical Physiology and Pharmacology 2020;30(6):20190247 | B |
| Bailey LC, Orosz Jr ST. Stability of ceftriaxone sodium and metronidazole hydrochloride. American Journal of Health-System Pharmacy. 1997;54(4):424-7. | D |
| Ballereau F, Prazuck T, Schrive I, Lafleuriel MT, Rozec D, Fisch A, et al. Stability of essential drugs in the field: Results of a study conducted over a two-year period in Burkina Faso. American Journal of Tropical Medicine and Hygiene. 1997;57(1):31-6. | D |
| Bansal D, Malla S, Gudala K, Tiwari P. Anti-counterfeit technologies: A pharmaceutical industry perspective. Scientia Pharmaceutica. 2013;81(1):1-13. | A |
| Baratta F, Germano A, Brusa P. Diffusion of counterfeit drugs in developing countries and stability of galenics stored for months under different conditions of temperature and relative humidity. Croat Med J. 2012;53(2):173–184. | B |
| Bate R, Jin GZ, Mathur A. Does price reveal poor-quality drugs? Evidence from 17 countries. J Health Econ. 2011;30(6):1150–1163. (corresponds to Working Paper 16854 http://www.nber.org/papers/w16854) | B |
| Bate R, Jin GZ, Mathur A. Falsified or substandard? Assessing price and non-price signals of drug quality. J Econ Manage Strategy. 2015;24(4):687-711. doi:10.1111/jems.12114 (corresponds to Quality‖NBER Working Paper No. 18073) | B |
| Bate R, Mooney L, Hess K, Milligan J, Attaran A. Anti-infective medicine quality: analysis of basic product quality by approval status and country of manufacture. Res Rep Trop Med. 2012 Jul 13;3:57-61 | E |
| Bate R, Mooney L, Hess K.Medicine registration and medicine quality: a preliminary analysis of key cities in emerging markets. Research and Reports in Tropical Medicine 2010:1 89–93 | B |
| Bate R, Tren R, Hess K, Mooney L, Porter K. Pilot study comparing technologies to test for substandard drugs in field settings. African Journal of Pharmacy and Pharmacology. 2009;3(4):165–170. | B |
| Bate R, Tren R, Mooney L, Hess K, Mitra B, Debroy B, Attaran A. Pilot study of essential drug quality in two major cities in India. PLoS ONE 2009; 4: e6003. https://www.ncbi.nlm.nih.gov/pmc/articles/PMC2695555/ | C |
| Bate, R.Mathur A. Corruption and Medicine Quality in Latin America: A Pilot Study. American Enterprise Institute | B |
| Behrens RH, Awad AI, Taylor RB. Substandard and counterfeit drugs in developing countries. Trop Doct 2002; 32: 1–2. | A |
| Belliveau PP, Nightingale CH, Quintiliani R. Stability of cefotaxime sodium and metronidazole in 0.9% sodium chloride injection or in ready-to-use metronidazole bags. American Journal of Health-System Pharmacy. 1995;52(14):1561-3. | D |
| Berard V, Fiala C, Cameron S, Bombas T, Parachini M, Gemzell-Danielsson K. Instability of misoprostol tablets stored outside the blister: A potential serious concern for clinical outcome in medical abortion. PLoS ONE. 2014;9(12):e112401. | D |
| Berard V, Fiala C. The effects of bad storage conditions on the quality and the related effectiveness of Cytotec. BJOG: An International Journal of Obstetrics and Gynaecology. 2012;119(SUPPL. 2):15-6. | D |
| Blume H, Ali SL, Siewert M. The quality of drugs from the new federal states of Germany. Pharmazeutische Zeitung. 1992;137(10):30-4. | F |
| Borner K, Reinauer H. External quality control of drug analyses: Results of a ring study in 1981-85. LaboratoriumsMedizin. 1986;10(9):244-8. | F |
| Brandl E, Gapp F, Knauseder F. Investigation of purity and storage stability of penicillin G sodium. Scientia Pharmaceutica. 1974;42(4):209-21. | F |
| Brhlikova P, Harper I, Jeffery R, Rawal N, Subedi M, Santhosh MR. Trust and the regulation of pharmaceuticals: South Asia in a globalised world. Globalization and Health. 2011;7:10. | A |
| Brits M, Kopp S. Combating unsafe medical products: Outcomes of a survey on testing of suspect medicines. WHO Drug Information. 2014;28(3):317-23. | A |
| Campos LM, Schapoval EE. Controle de qualidade de produtos farmaceuticos contendo gentamicina. An farm quím Säo Paulo. 1982;22(1/2):27-33. | G |
| Carpenter J. A review of drug quality in 11 Asian countries with focus on anti-infectives. United States Pharmacopoeia (USP), Drug Quality and Information Program, February 2004, pp 1– 46. Available at: http://www.uspdqi.org/pubs/other/ANEReview.pdf. | A |
| Caudron JM, Ford N, Henkens M, Mace C, Kiddle-Monroe R, Pinel J. Substandard medicines in resource-poor settings: A problem that can no longer be ignored. Tropical Medicine and International Health. 2008;13(8):1062-72. | A |
| CDSCO. Report on Countrywide Survey for Spurious Drugs. Central Drugs Standrards Control Organisation, Directorate General of Health Services, . Ministry of Health and Family Welfare. Government Of India, 2009. http://apps.who.int/medicinedocs/documents/s19288en/s19288en.pdf | B |
| Charnock C. The microbial content of non-sterile pharmaceuticals distributed in Norway. Journal of Hospital Infection. 2004;57(3):233-40. | F |
| Chaudhury RR, Parameswar R, Gupta U, Sharma S, Tekur U, Bapna JS. Quality medicines for the poor: Experience of the Delhi programme on rational use of drugs. Health Policy and Planning. 2005;20(2):124-36. | A |
| Chunyang SHI, Jiamin DU, Mengdie W, Jinli C, Zhiwei YE, Jianguo F, et al. Similarity Investigation on Dissolution Curves of Metronidazole Tablets from Different Manufacturers. Herald of Medicine. 2017:917-22. | C |
| Cobos Campos R, Salvador Collado P, Gomez Gener A, Boj Borbones M. Maximum stability of thermolabile drugs outside the refrigerator. Farmacia Hospitalaria. 2006;30(1):33-43. | F |
| Cohen HE. Fake drugs are real. US Pharmacist. 2010;35(12):3. | A |
| Cohen V, Jellinek SP, Teperikidis L, Berkovits E, Goldman WM. Room-temperature storage of medications labeled for refrigeration. American Journal of Health-System Pharmacy. 2007;64(16):1711-5. | A |
| Conway J, Bero L, Ondari C, Wasan KM. Review of the quality of pediatric medications in developing countries. Journal of Pharmaceutical Sciences. 2013;102(5):1419-33. | A |
| Craig DB, Martens DJ, Embil JM. Single dose medication vial packaging deficiencies. Canadian Journal of Anaesthesia. 1998;45(6):501-8. | A |
| Crichton B. Keep in a cool place: Exposure of medicines to high temperatures in general practice during a British heatwave. Journal of the Royal Society of Medicine. 2004;97(7):328-9. | A |
| Cuervas-Mons Vendrell M, Fernandez Prieto M, Sanchez Sanchez MT, Maestre Fullana MA, Abad Lecha E, Salvador Palacios A, et al. Potential validity of thermolabile drugs outside the preservation conditions recommended by the manufacturer. Farmacia Hospitalaria. 2004;28(6):440-4. | A |
| Dammertz W, Paulus H. Quantitative TLC for quality control of drugs stored long-term by the German Federal Armed Forces Medical Service. Journal of Planar Chromatography - Modern TLC. 1995;8(4):314-8. | G |
| Dégardin K, Roggo Y, Margot P. Understanding and fighting the medicine counterfeit market. J Pharm Biomed Anal. 2014 Jan;87:167-75. | A |
| Deisingh AK. Pharmaceutical counterfeiting. Analyst 2005; 130: 271–79. | A |
| Delepierre A, Gayot A, Carpentier A. Update on counterfeit antibiotics worldwide; public health risks. Med Mal Infect. 2012 Jun;42(6):247-55. | A |
| Derharoutunian C, Roubille R, Bec JF, Bochaton C, Rochegude S, Galtier H. Emergency drug stability at extreme temperatures. Pharmacie Hospitaliere Francaise. 1996(117 SPEC. ISS.):16-7. | A |
| Diop A, Sarr SO, Diop YM, Ndiaye B, Fall M, Mbaye G. [Quality control of antibiotics used in Senegal]. Medecine tropicale : revue du Corps de sante colonial. 2009;69(3):251-254. | C |
| Diven DG, Bartenstein DW, Carroll DR. Extending shelf life just makes sense. Mayo Clinic Proceedings. 2015;90(11):1471-4. | A |
| Ejekam CS, Okafor IP, Anyakora CA, Nwokike JI, Ozomata E, Okunade K, et al. Clinical experience with oxytocin quality used by health care providers in Lagos, South-West Nigeria: A cross-sectional study. Pharmacoepidemiology and Drug Safety. 2018;27(Supplement 2):440. | A |
| Frimpong G, Ofori-Kwakye K, Kuntworbe N, Buabeng KO, Osei YA, El Boakye-Gyasi M, et al. Quality Assessment of Some Essential Children's Medicines Sold in Licensed Outlets in Ashanti Region, Ghana. Journal of Tropical Medicine. 2018;2018:1494957. | B |
| Frye, L. J.; Kilfedder, C.; Blum, J.; Winikoff, B. A cross-sectional analysis of mifepristone, misoprostol, and combination mifepristone-misoprostol package inserts obtained in 20 countries Contraception 2020;101(5):315-320 | A |
| G.N. Thoithi, I.O. Kibwage, O. Kingondu and J. Hoogmartens. Liquid Chromatographic Separation of Isoniazid, Pyrazinamide and Rifampicin on a Reversed-Phase Silica Column East Cent. Afr. J. Pharm. Sci. 5(2002) 8-14. | B |
| Gadalla MAF, Ebian AR, Ghaly GM. Evaluation of commercial metronidazole tablets. Drug Development and Industrial Pharmacy. 1984;10(7):1097-115. | C |
| Gensthaler BM. Drug counterfeiting: Vigilance is top priority. Pharmazeutische Zeitung. 2004;149(39):18-22. | B |
| Gyanwali P, Humagain BR, Aryal KK, et al. Surveillance of Quality of Medicines Available in the Nepalese Market: A Study from Kathmandu Valley. Journal of Nepal Health Research Council. 2015;13(31):233-240. | B |
| Hadi U, van den Broek P, Kolopaking EP, Zairina N, Gardjito W, Gyssens IC. Cross-sectional study of availability and pharmaceutical quality of antibiotics requested with or without prescription (over the counter) in Surabaya, Indonesia. BMC Infect Dis 2010; 10: 203. | B |
| Hajjou M, Krech L, Lane-Barlow C, Roth L, Pribluda VS, Phanouvong S, El-Hadri L, Evans L, Raymond C, Yuan E, Siv L, Vuong TA, Boateng KP, Okafor R, Chibwe KM, Lukulay PH. Monitoring the quality of medicines: results from Africa, Asia, and South America. Am J Trop Med Hyg. 2015 Jun;92(6 Suppl):68-74. doi: 10.4269/ajtmh.14-0535. Epub 2015 Apr 20. | B |
| Hand RM, Senarathna G, Page-Sharp M, Sika-Paotonu D, Gray K, Batty KT, et al. Benzathine Penicillin G Quality and Potency - Do We Need a New Manufacturing Standard? Global Heart. 2018;13(4):519. | E |
| Hapsari, I.; Marchaban,; Wiedyaningsih, C.; Kristina, S. A. Microbial contamination on dosage form of non-sterile semi-solid extemporaneous compounding in primary health care centers International Medical Journal 2019;24(3):317-324 | C |
| Harper, J. 2006. Counterfeit Medicines Survey Report, Council for Europe. Strasbourg: Council of Europe Publishing. | F |
| Hetzel MW, Page-Sharp M, Bala N, Pulford J, Betuela I, Davis TM, Lavu EK. Quality of antimalarial drugs and antibiotics in Papua New Guinea: a survey of the health facility supply chain. PLoS One. 2014 May 14;9(5) | B |
| Hildebrand JR. Counterfeit drugs in the United States. Drugs Made in Germany. 2002;45(1):14-6. | A |
| Ho NT, Desai D, Fernandes A, Krull W, Banahene A, Togoh G, et al. Testing drug quality at all points in the supply chain: Integration of technology and health system. American Journal of Tropical Medicine and Hygiene. 2015;93(4 Supplement):211. | E |
| Hodgins S, Lukulay PH. The impact of thermal stability of oxytocin on access, and the importance of setting proper product specifications. International Journal of Gynecology and Obstetrics. 2017;136(3):253-4. | A |
| Hogerzeil HV, Battersby A, Srdanovic V, et al. WHO/UNICEF study on stability of drugs during international transport. WHO/DAP/91 1. Geneva: World Health Organisation, 1991. | D |
| Hogerzeil HV, Battersby A, Srdanovic V, Stjernstrom NE. Stability of essential drugs during shipment to the tropics. British Medical Journal. 1992;304(6821):210-4. | D |
| Hogerzeil HV, de Goeje M, Abu Reid 10. Inland Stability Study (Sudan): Pilot Study 1989-1991. WHO/DAP/91.4. Geneva: WHO, 1991 | D |
| Hogerzeil HV, De Goeje MJ, Abu-Reid IO. Stability of essential drugs in Sudan [2]. Lancet. 1991;338(8769):754-5. | E |
| Hogerzeil HV, Walker GJA, De Goeje MJ. Managing the third stage of labour: Oxytocin more stable in tropical climates [9]. British Medical Journal. 1994;308(6920):59. | D |
| Hogerzeil HV, Walker GJA, Van de Langerijt AEEM. The colour of ergometrine injection: How to recognize low level of active ingredient. Tropical Doctor. 1994;24(3):112-4. | A |
| Hogerzeil HV, Walker GJA. Instability of (methyl)ergometrine in tropical climates: An overview. European Journal of Obstetrics Gynecology and Reproductive Biology. 1996;69(1):25-9. | D |
| Hollein L, Kaale E, Mwalwisi YH, Schulze MH, Holzgrabe U. Routine quality control of medicines in developing countries: Analytical challenges, regulatory infrastructures and the prevalence of counterfeit medicines in Tanzania. TrAC - Trends in Analytical Chemistry. 2016;76:60-70. | A |
| Höllein, L., Kaale, E., Mwalwisi, Y. H, Schulze, M. H, & Holzgrabe, U. (2016). Routine quality control of medicines in developing countries: Analytical challenges, regulatory infrastructures and the prevalence of counterfeit medicines in Tanzania. Trends in analytical chemistry, 76, 60-70. | A |
| Holzgrabe U, Deubner R, Wienen F. Quality of APIs - Result of a research of different gentamicin products. American Pharmaceutical Outsourcing. 2004;5(3):24-8. | G |
| Holzgrabe U. Quality of APIs - Gentamicin and others. American Pharmaceutical Outsourcing. 2007;8(2). | G |
| How stable are medicines moved from original packs into compliance aids? The Pharmaceutical Journal 21 JAN 2006 https://www.pharmaceutical-journal.com/news-and-analysis/how-stable-are-medicines-moved-from-original-packs-into-compliance-aids/10990832.article?firstPass=false | A |
| Hu CQ, Zou WB, Hu WS et al. Establishment of a fast chemical identification system for screening of counterfeit drugs of macrolide antibiotics. J Pharm Biomed Anal 2006; 40: 68–74. | B |
| Ibezim EC, Esimone CO, Ofoefule SI, Chah KF. Evaluation of the microbiological quality of some commercially available syrups and suspensions in Nigeria. Journal of Phytomedicine and Therapeutics. 2002;7(1-2):18-25. | C |
| Indrayani, Harianis S, Astuti H, Rosmaria. How is oxytocin cold chain in peripheral areas and is it still effective uterotonic? Pakistan Journal of Medical and Health Sciences. 2018;12(4):1744-9. | A |
| Issack MI. Substandard drugs. Lancet 2001; 358: 1463. | B |
| Itah AY, Udokpoh AE, Ofum MU. Bacteriological quality of some pharmaceutical products marketed by drug vendors in Uyo, Nigeria. African journal of health sciences. 2004;11(3-4):128-33. | C |
| Jackson G, Patel S, Khan S. Assessing the problem of counterfeit medications in the United Kingdom. International Journal of Clinical Practice. 2012;66(3):241-50. | F |
| Joshi, S. S.; Shetty, Y. C.; Karande, S. Generic drugs - The Indian scenario Journal of Postgraduate Medicine 2019;65(2):67-69 | A |
| K.O. Abuga, P.M. Mwagiru, G.N. Thoithi, J.M. Nguyo, J.K. Ngugi, O.K. King’ondu, H.N. Mugo and I.O. Kibwage.Quality of Antiretroviral Drugs Analyzed in the Drug Analysis and Research Unit During 2000-2003 . East Cent. Afr. J. Pharm. Sci. 6 (2003) 20-23 | B |
| Kadima JN, Nyandwi JB, Kayitana CI, Mashaku A. Assessing Pharmaceutical Equivalence of Generic Antibiotics Using in vitro Antimicrobial Susceptibility of Some Hospital Strains in Rwanda. Br J Med Med Res. 2016. | C |
| Kahaliw W, Ashenef A. Comparative quality evaluation of some metronidazole tablets and metronidazole benzoate oral suspensions available in retail outlets of addis ababa, ethiopia. International Journal of Pharmaceutical Sciences and Research. 2013;4(4):1384-91. | C |
| Karlage KL, Franklin SJ, Mufich WC, Goetz KJ, Sabelka JV, Hoye WL, et al. Comparative evaluation of pharmaceutical products obtained in Mexico: Augmenting existing scientific data. Drug Development and Industrial Pharmacy. 2012;38(7):808-14. | C |
| Karlage KL, Myrdal PB. (2005). Comparison of three pharmaceutical products obtained from Mexico and the United States: A case study. Drug Dev Ind Pharm, 31:993–1000. | B |
| Kartoglu U, Widmer M, Gulmezoglu M. Stability of oxytocin along the supply chain: A WHO observational study. Biologicals. 2017;50:117-24. | D |
| Kaur, H., 2015. Findings from the ACT Consortium drug quality programme in 6 countries. http://www.actconsortium.org/data/files/9_Harparkash_Kaur_Field_work_final.pdf | B |
| Kayumba PC, Risha PG, Shewiyo D, Msami A, Masuki G, Ameye D, et al. The quality of essential antimicrobial and antimalarial drugs marketed in Rwanda and Tanzania: Influence of tropical storage conditions on in vitro dissolution. Journal of Clinical Pharmacy and Therapeutics. 2004;29(4):331-8. | C |
| Kelesidis T, Falagas ME. Substandard/counterfeit antimicrobial drugs. Clinical Microbiology Reviews. 2015;28(2):443-64. | A |
| Kelesidis T, Kelesidis I, Rafailidis PI, Falagas ME. Counterfeit or substandard antimicrobial drugs: A review of the scientific evidence. Journal of Antimicrobial Chemotherapy. 2007;60(2):214-36. | A |
| Khan AN, Khar RK. Current scenario of spurious and substandard medicines in india: A systematic review. Indian Journal of Pharmaceutical Sciences. 2015;77(1):2-7. | C |
| Khan MH, Okumura J, Sovannarith T, Nivanna N, Nagai H, Taga M, et al. Counterfeit medicines in Cambodia - Possible causes. Pharmaceutical Research. 2011;28(3):484-9. | A |
| Khojah, HMJ, Pallos, H, Yoshida, N (2013) “The Quality of Medicines in Community Pharmacies in Riyadh, Saudi Arabia: A Lot Quality Assurance Sampling (LQAS)-Based Survey,” Pharmacology & Pharmacy, 4:511-19. | B |
| Khuluza F, Kigera S, Heide L. Low prevalence of substandard and falsified antimalarial and antibiotic medicines in public and faith-based health facilities of southern Malawi. American Journal of Tropical Medicine and Hygiene. 2017;96(5):1124-35. | B |
| Khurelbat D, Dorj G, Bayarsaikhan E, et al. Prevalence estimates of substandard drugs in Mongolia using a random sample survey. Springerplus. 2014;3:709. doi:10.1186/2193-1801-3-709 | C |
| Kibwage IO, Thuranira J, Migosi D. Quality performance of metronidazole tablet products on the Kenyan market. East African medical journal. 1991;68(5):365-71. | C |
| Kitutu FE; Uganda Medicines Transparency Alliance. Screening Drug Quality Project Report. Kampala, Uganda: Uganda Medicines Transparency Alliance; 2015.http://apps.who.int/medicinedocs/documents/s22323en/s22323en.pdf | B |
| Krech LA, El-Hadri L, Evans L, Fouche T, Hajjou M, Lukulay P, Phanouvong S, Pribluda V, Roth L. The Medicines Quality Database: a free public resource. Bull World Health Organ. 2014 Jan 1;92(1):2-2A. | A |
| Krieger JA, Duncan L. Gentamicin contaminated with endotoxin [6]. New England Journal of Medicine. 1999;340(14):1122. | F |
| Lehmann A, Hofsäss M, Dressman J. Differences in drug quality between South Africa and Germany. J Pharm Pharmacol. 2018 Oct;70(10):1301-1314. doi: 10.1111/jphp.12985. | B |
| Lehmann A, Katerere DR, Dressman J. Drug Quality in South Africa: A Field Test. J Pharm Sci. 2018 Oct;107(10):2720-2730. doi: 10.1016/j.xphs.2018.06.012. | B |
| Lobenberg R, Chacra NB, Stippler ES, Shah VP, Destefano AJ, Hauck WW, et al. Toward global standards for comparator pharmaceutical products: Case studies of amoxicillin, metronidazole, and zidovudine in the Americas. AAPS Journal. 2012;14(3):462-72. | C |
| Lybecker K. Rx roulette: counterfeit pharmaceuticals in developing nations, 2003. http://www.lebow.drexel.edu/lybecker/Lybecker.pdf | A |
| Mack, I.; Sharland, M.; Rehm, S.; Rentsch, K.; Bielicki, J. Clavulanate stability in child-appropriate formulations is inadequate for use in treating young children in Asia Archives of Disease in Childhood 2019;104(6): | B |
| Matrix of Drug Quality Reports Affecting USAID-assisted Countries By the U.S. Pharmacopeia Drug Quality and Information Program 2008 | A |
| Matrix of Drug Quality Reports Affecting USAID-assisted Countries By the U.S. Pharmacopeia Drug Quality and Information Program 2009 | A |
| Matrix of Drug Quality Reports in USAID-assisted Countries By the U.S. Pharmacopeia Drug Quality and Information Program 2006  http://www.uspdqi.org/pubs/other/GHC-DrugQualityMatrix.pdf | A |
| Mbaziira N. Registration and Quality Assurance of ARVs & Other Essential Medicines in Namibia: October 2014- September 2015. Arlington, VA: Management Sciences for Health; 2015 http://apps.who.int/medicinedocs/documents/s22456en/s22456en.pdf | B |
| Medicine Quality Monitoring Program 5-year Report Cambodja 2005-2009 | C |
| Medicine quality monitoring program in the Mekong region (2009). U.S. Pharmacopeia Drug Quality and Information (USP DQI) Program https://www.usp-pqm.org/sites/default/files/pqms/article/the-state-of-medicines-quality-in-the-mekong-subregion.pdf | E |
| Medicines Quality Monitoring Program 5-year Report Viet Nam 2005 - 2010 | B |
| Menkes DB. Hazardous drugs in developing countries. BMJ 1997; 315: 1557–8. | A |
| Mori AT, Meena E, Kaale EA. Economic cost of substandard and falsified human medicines and cosmetics with banned ingredients in Tanzania from 2005 to 2015: A retrospective review of data from the regulatory authority. BMJ Open. 2018;8(6):e021825. | A |
| Morris, J., Stevens, P., & International Policy Network. (2006). Counterfeit medicines in less developed countries: Problems and solutions. London, England: International Policy Network. | A |
| Musa H, Sule YZ, Gwarzo MS. Assessment of physicochemical properties of metronidazole tablets marketed in Zaria, Nigeria. International Journal of Pharmacy and Pharmaceutical Sciences. 2011;3(SUPPL. 3):27-9. | C |
| Nagaraj A, Tambi S, Shravani G, Biswas G, Kumawat H, Mathur G. Counterfeit medicines sale on online pharmacies in India. Journal of Research in Pharmacy Practice. 2014;3(4):145-6. | B |
| Naimi RKA, Khan SA. Comparative in-vitro pharmaceutical evaluation of four brands of Metronidazole tablets marketed in gulf region. Jordan Journal of Pharmaceutical Sciences. 2014;7(2):144-52. | C |
| Nair A, Strauch S, Lauwo J, Jähnke RW, Dressman J. Are counterfeit or substandard anti-infective products the cause of treatment failure in Papua New Guinea? J Pharm Sci. 2011;100(11):5059–5068. | B |
| Naveed S, Mateen N, Nazeer S. Degradation studies of Ampicillin in API and formulations. Journal of Applied Pharmacy. 2014;6(3):314-21. | D |
| Newton PN, Green MD, Fernandez FM, Day NP, White NJ. Counterfeit anti-infective drugs. Lancet Infectious Diseases. 2006;6(9):602-13. | A |
| Newton PN, Green MD, Fernández FM. Impact of poor-quality medicines in the 'developing' world. Trends Pharmacol Sci. 2010 Mar;31(3):99-101. | A |
| Newton PN, Tabernero P, Dwivedi P, Culzoni MJ, Monge ME, Swamidoss I, Mildenhall D, Green MD, Jähnke R, de Oliveira MDS, Simao J, White NJ, Fernández FM. Falsified medicines in Africa: all talk, no action. Lancet Glob Health. 2014 Sep;2(9):e509-e510 | A |
| Newton, P.N. et al., 2010. Counterfeit and substandard anti-infectives in developing countries. In Antimicrobial Resistance in Developing Countries. Springer, pp. 413–443. | A |
| Nguyen TH, Lambert P, Minhas RS, McEvoy C, Deadman K, Wright P, Prankerd RJ, Mogatle S, McIntosh MP. Temperature stability of oxytocin ampoules labelled for storage at 2°C-8°C and below 25°C: an observational assessment under controlled accelerated and temperature cycling conditions. BMJ Open. 2019 Jul 26;9(7):e029083. | D |
| Nicholas E, Hess G, Colten HR. Degradation of penicillin, ticarcillin, and carbenicillin resulting from storage of unit doses. New England Journal of Medicine. 1982;306(9):547-8. | D |
| Nkang AO, Okonko IO, Lennox JA, Eyarefe OD, Abubakar MJ, Ojezele MO, et al. Assessment of the efficacies, potencies and bacteriological qualities of some of the antibiotics sold in Calabar, Nigeria. African Journal of Biotechnology. 2010;9(41):6987-7002. | A |
| Nwokike J, Clark A, Nguyen PP. Medicines quality assurance to fight antimicrobial resistance. Bull World Health Organ. 2018 Feb 1;96(2):135-137 | A |
| Obodozie OO, Mustapha KB, Ebeshi BU, Inyang US. A comparative study on the prevalence of substandard ampicillin/cloxacillin preparations in the Nigerian market: Mid 1990's and present. Journal of Phytomedicine and Therapeutics. 2006;8-11:1-8. | B |
| Okumura J, Taga M, Tey S, Kataoka Y, Nam N, Kimura K. High failure rate of the dissolution tests for 500-mg amoxicillin capsules sold in Cambodia: is it because of the product or the test method? Trop Med Int Health. 2010 Nov;15(11):1340-6. | B |
| Oldenhof C. APIs: Why EU authority oversight is vital. Pharmaceutical Technology Europe. 2006;18(3):32-6. | A |
| Omer AIH, Stjernstrom NE. Stability of drugs in the tropics. A study in Sudan. Tropical Doctor. 1990;20(3):129. | E |
| Ononna HF, Al Hossain ASMM, Ganguly A, Faroque ABM. Potency determination of essential drug samples manufactured by small and medium pharmaceutical industries in Bangladesh. Bangladesh Medical Research Council Bulletin. 2017;43(2):87-93. | C |
| Osei-Safo, D; Egbo, HA; Nettey, H; et al. (2016) “Evaluation of the Quality of Some Antibiotics Distributed in Accra and Lagos,” International Journal of Pharmaceutical Sciences and Research, 7(5):1991-2000. | C |
| Pan H, Luo H, Chen S et al. Pharmacopoeial quality of antimicrobial drugs in southern China. Lancet Glob Health 2016; 4: e300–2. | C |
| Patel A, Gauld R, Norris P, Rades T. Quality of generic medicines in South Africa: perceptions versus reality—a qualitative study. BMC Health Serv Res. 2012;12(1):297. doi:10.1186/1472-6963-12-297 | B |
| Paterson and Karimi, 2005. “Understanding markets in Afghanistan: A study of the market of Pharmaceuticals”, available at  https://areu.org.af/wp-content/uploads/2015/12/532E-Market-for-Pharmaceuticals-CS-web.pdf | A |
| Perks, S. J.; Lanskey, C.; Robinson, N.; Pain, T.; Franklin, R. Systematic review of stability data pertaining to selected antibiotics used for extended infusions in outpatient parenteral antimicrobial therapy (OPAT) at standard room temperature and in warmer climates European Journal of Hospital Pharmacy 2020;27(2):65-72 | A |
| Peters, D., A. A. Noor, L.P. Singh, et al. 2007. A balanced scorecard for health services in Afghanistan. Bulletin of the World Health Organization 85:146-151. | A |
| Petersen A, Held N, Heide L. Surveillance for falsified and substandard medicines in Africa and Asia by local organizations using the low-cost GPHF Minilab. PLoS ONE. 2017;12(9):e0184165. | C |
| Pharmaceutical and vaccine quality ILLUSTRATED 2015 ISBN 978-2-9701065-2-4 | A |
| Pichini S, Rotolo MC, Bellotti P, Minutillo A, Mastrobattista L, Pacifici R. Quali-quantitative analysis of best selling drugs from pharmacy, street market and traditional herbal medicine: a pilot study of market surveillance in Senegal. Journal of pharmaceutical and biomedical analysis.2015;104:62-66 | B |
| Pierrot Mwamba T, Duez P, Jean Baptiste Kalonji N. Preliminary survey of counterfeiting of albendazole and metronidazole marketed in Lubumbashi. International Journal of Pharmacy and Pharmaceutical Sciences. 2016;8(7):282-7. | C |
| Po ALW. Too much, too little, or none at all: Dealing with substandard and fake drugs. Lancet. 2001;357(9272):1904. | E |
| Pouillot R, Bilong C, Boisier P, et al. Le circuit informel des médicaments à Yaoundé et à Niamey : étude de la population des vendeurs et de la qualité des médicaments distribués. [Illicit drug trade on the markets of Yaounde (Cameroon) and Niamey (Niger): characteristics of salesmen and quality of drugs]. Bull Soc Pathol Exot. 2008;101(2):113–118. | E |
| Ratprasatporn, N.; Wittayalertpanya, S.; Khemsri, W.; Chatsuwan, T.; Chongpison, Y.; Chamsai, T.; Wattanakijkarn, M.; Chansangpetch, S. Stability and Sterility of Extemporaneously Prepared Nonpreserved Cefazolin, Ceftazidime, Vancomycin, Amphotericin B, and Methylprednisolone Eye Drops. Cornea 2019;38(8):1017-1022 | C |
| Ravinetto RM, Boelaert M, Jacobs J, Pouget C, Luyckx C. Poor-quality medical products: time to address substandards, not only counterfeits. Trop Med Int Health. 2012 Nov;17(11):1412-6. doi: 10.1111/j.1365-3156.2012.03076.x. (Editorial) | A |
| Risha PG, Msuya Z, Clark M, Johnson K, Ndomondo-Sigonda M, Layloff T. The use of Minilabs to improve the testing capacity of regulatory authorities in resource limited settings: Tanzanian experience. Health Policy. 2008;87(2):217–222. | E |
| Risha PG, Shewiyo D, Msami A, et al. In vitro evaluation of the quality of essential drugs on the Tanzanian market. Tropical medicine & international health : TM & IH. 2002;7(8):701-707. | B |
| Roger Bate, Ginger Zhe Jin ,Aparna Mathur. Report. Counterfeit or substandard? Assessing Price and Non-Price signals of drug quality. Working Paper 18073 http://www.nber.org/papers/w18073 | B |
| Roger Bate, Ginger Zhe Jin, Aparna Mathur, Amir Attaran. Poor quality drugs and global trade: a pilot study. Working Paper 20469 http://www.nber.org/papers/w20469. | B |
| Roy J. The menace of substandard drugs. World Health Forum 1994; 15: 406–7 | C |
| Rudolf PM, Bernstein IB. Counterfeit drugs. N Engl J Med 2004; 350: 1384–86. | A |
| Schafermann S, Wemakor E, Hauk C, Heide L. Quality of medicines in southern Togo: Investigation of antibiotics and of medicines for non-communicable diseases from pharmacies and informal vendors. PLoS ONE. 2018;13(11):e0207911. | C |
| Schwertner HA, Storrow AB. (2005). Comparison of actual and stated concentrations of pharmaceuticals manufactured in Mexico. Clin Ther, 27:609–615. | B |
| Seear M, Gandhi D, Carr R, Dayal A, Raghavan D, Sharma N. The need for better data about counterfeit drugs in developing countries: a proposed standard research methodology tested in Chennai, India. J Clin Pharm Ther. 2011;36(4):488–495. | B |
| Seema Thakral, Raj Suryanarayanan, Lawrence Evans, Paul Nkansah. Revisiting the Stability and Storage Specifications of Oxytocin Injection Formulation: A Literature Review. 2018. U.S. Pharmacopeial Convention. The Promoting the Quality of Medicines Program. Rockville, Maryland. https://www.usp-pqm.org/sites/default/files/pqms/article/stability-storage-oxytocin-jul2018.pdf | A |
| Shakoor O, Taylor RB, Behrens RH. Assessment of the incidence of substandard drugs in developing countries. Trop Med Int Health 1997; 2: 839–45. | B |
| Shepherd M. Vulnerable points in the U.S. drug-distribution system. Manag Care. 2004 Mar;13(3 Suppl):25-9. | A |
| Sillo HB, Masota NE, Kisoma S, Rago L, Mgoyela V, Kaale EA. Conformity of package inserts information to regulatory requirements among selected branded and generic medicinal products circulating on the East African market. PLoS ONE. 2018;13(5):e0197490. | C |
| Singal, GL, Nanda, A, Kotwani, A (2011) “A comparative evaluation of price and quality of some branded versus branded–generic medicines of the same manufacturer in India,” Indian Journal of Pharmacology, 43(2):131-36. | B |
| Singh BK, Parwate DV, Shukla SK. Screening of counterfeit cephalosporin and discrimination from penicillins by high-throughput chemical color tests. PDA Journal of Pharmaceutical Science and Technology. 2010;64(2):97-100. | G |
| Singhal GL, Anita K, Nanda A. Jan Aushadhi stores in India and quality of medicines therein. International Journal of Pharmacy and Pharmaceutical Sciences. 2011;3(1):204-207 | B |
| Sow PS, Gueye TS, Sy E et al. Drugs in the parallel market for the treatment of urethral discharge in Dakar: epidemiologic investigation and physicochemical tests. Int J Infect Dis 2002; 6: 108–12. | C |
| Stenson B, Lindgren BH, Syhakhang L, Tomson G. The quality of drugs in private pharmacies in the Lao People’s Democratic Republic. Int J Risk Safety Med 1998; 11: 243–49. | C |
| Stewart AG. Expiry dates on drug packages [2]. Tropical Doctor. 1997;27(3):191. | E |
| Sumon S, Aporanee C, Suthep W, Detpon P, Chantana A, Thanee T, et al. A Survey on Qualities of Drugs Commercially Avilable in Thailand. 2010. | C |
| Sunenshine, R.H. et al 2007. A multistate outbreak of Serratia marcescens bloodstream infection associated with contaminated intravenous magnesium sulfate from a compounding pharmacy. Clin. Inf. Dis. 45:527–33. https://academic.oup.com/cid/article/45/5/527/273668 | F |
| Syhakhang L, Lundborg CS, Lindgren B, Tomson G. The quality of drugs in private pharmacies in Lao PDR: a repeat study in 1997 and 1999. Pharm World Sci 2004; 26: 333–8. | C |
| Syhakhang L. The Quality of Private Pharmacy Services in a Province of Lao PDR: Perceptions, Practices and Regulatory Enforcements. Stockholm, Sweden: Karolinksa Institutet, Division of International Health, Department of Public Health Sciences; 2002. Syhakhang L. The Quality of Private Pharmacy Services in a Province of Lao PDR: Perceptions, Practices and Regulatory Enforcements. Stockholm, Sweden: Karolinksa Institutet, Division of International Health, Department of Public Health Sciences; 2002. | C |
| Tie, Y.; Adams, E.; Deconinck, E.; Vanhee, C. Substandard and falsified antimicrobials: A potential biohazard in disguise? Drug Testing and Analysis 2020;12(2):285-291 | F |
| Tie, Y.; van Loock, K.; Deconinck, E.; Adams, E. Evaluation of impurities and dissolution profiles of illegal antimicrobial drugs encountered in Belgium. Drug Testing and Analysis 2020;12(1):53-66 | F |
| Tomic S, Filipovic-Sucic A. Counterfeit medicines. Pharmaca. 2009;47(1-2):3-16. | I |
| Tomlinson R. China cracks down on counterfeit medicines BMJ 1999;318:624 | A |
| Tshilumba PM, Amuri SB, Kaghowa ER, et al. Enquête sur la contrefaçon de quelques anti-infectieux administrés per os commercialisés dans la ville de Lubumbashi. Pan Afr Med J. 2015;22:318. doi:10.11604/pamj.2015. 22.318.7302. http://www.panafrican-med-journal.com/content/article/22/318/full/ | C |
| Uganda Medicines Transparency Alliance. Screening Drug Quality Project Report. Kampala, Uganda: Uganda Medicines Transparency Alliance; 2014 http://apps.who.int/medicinedocs/en/m/abstract/Js22322en/ | B |
| Wafula F, Dolinger A, Daniels B, Mwaura N, Bedoya G, Rogo K, et al. Examining the Quality of Medicines at Kenyan Healthcare Facilities: A Validation of an Alternative Post-Market Surveillance Model That Uses Standardized Patients. Drugs - Real World Outcomes. 2017;4(1):53-63. | C |
| Wang, S.W. Hoag, M.L. Eng, J. Polli, N.S. Pandit, Quality of antiretroviral and opportunistic infection medications dispensed from developing countries and Internet pharmacies, J. Clin. Pharm. Ther. 40 (2015) 68–75. https://onlinelibrary.wiley.com/doi/abs/10.1111/jcpt.12226 | B |
| Watts J. More on the risks of inconsistent drug packaging and ampoule labelling. Anaesthesia. 2016;71(3):348-9. | A |
| Wertheimer AI, Norris J. Safeguarding against substandard/counterfeit drugs: mitigating a macroeconomic pandemic. Res Social Adm Pharm. 2009 Mar;5(1):4-16 | A |
| WHO Counterfeit and Substandard Drugs in Myanmar and Viet Nam - Report of a Study Carried out in Cooperation with the Governments of Myanmar and Viet Nam - EDM Research Series N0. 029 (1999; 55 pages) . DAP 99.3 http://apps.who.int/medicinedocs/en/d/Js2276e/7.3.html#Js2276e.7.3 | B |
| WHO. Accelerated Stability Studies of Widely Used Pharmaceutical Substances Under Simulated Tropical Conditions. 1986: 119. Geneva:WHO/PHARM/86.529 https://apps.who.int/iris/bitstream/handle/10665/61480/WHO_PHARM_86.529.pdf?sequence=1&isAllowed=y | D |
| World Health Organization. Substandard/Spurious/Falsely-Labelled/Falsified/Counterfeit Medical Products: Report of the Working Group of Member States. 2012. Available online: http://apps.who.int/gb/ebwha/pdf_files/WHA65/A65_23-en.pdf (accessed on 28 July 2018). | A |
| Yang D, Plianbangchang P, Visavarungroj N, Rujivipat S. Quality of pharmaceutical items available from drugstores in Phnom Penh, Cambodia. Southeast Asian J Trop Med Public Health. 2004 Sep;35(3):741-7. | B |
| Yong YL, Plancon A, Lau YH, Hostetler DM, Fernandez FM, Green MD, et al. Collaborative health and enforcement operations on the quality of antimalarials and antibiotics in southeast Asia. The American journal of tropical medicine and hygiene. 2015;92(6 Supplement):105-12. | C |
| Yoshida, N, Khan, MH, Tabata, H (2014) A cross-sectional investigation of the quality of selected medicines in Cambodia in 2010. BMC Pharmacology and Toxicology, 15(13). | B |
