# Supplementary material for: Quality of medicines for life-threatening pregnancy complications in low- and middle-income countries: A systematic review
Source: PLoS One. 2020 Jul 10;15(7):e0236060. doi: 10.1371/journal.pone.0236060 (PMC7351160; doi:10.1371/journal.pone.0236060)
Supplement: S1 Fig — (DOCX) [file pone.0236060.s004.docx]

**S1 Figure. Distribution of quality scores^1^ of studies over time**


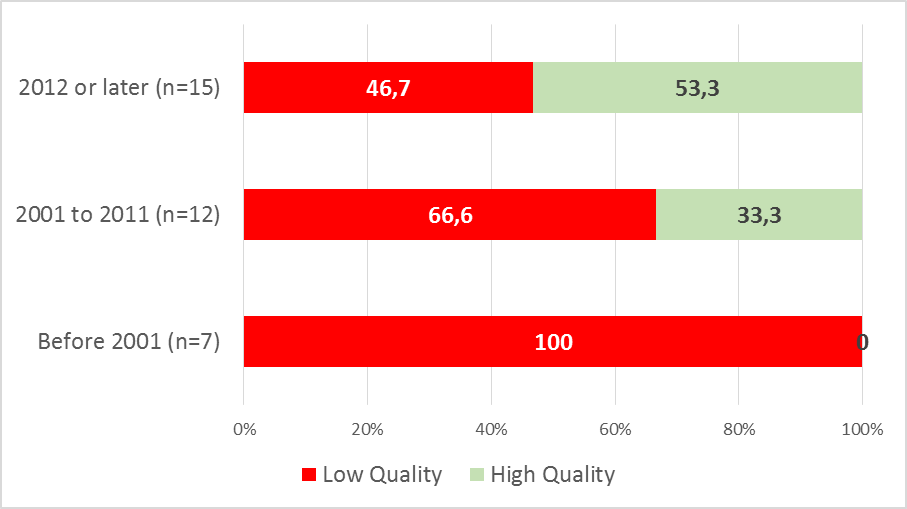


Numbers in parentheses indicate the total number of studies in each period.

Low quality: total quality score < 6. High quality: total quality score > 6.

1. Based on MEDQUARG guidelines 12 Quality Criteria (Newton 2009, Almuzaini 2013)
