# Supplementary material for: Quality of medicines for life-threatening pregnancy complications in low- and middle-income countries: A systematic review
Source: PLoS One. 2020 Jul 10;15(7):e0236060. doi: 10.1371/journal.pone.0236060 (PMC7351160; doi:10.1371/journal.pone.0236060)
Supplement: S1 Table — (DOCX) [file pone.0236060.s005.docx]

**S1 Table. Main details of 34 studies included in systematic review**

| **Study** | **Country** | **Country income** | **Year^1^ of sample collection** | **Total number of samples collected and tested** | **Number and type of samples per medicine** | **Tests Performed** | **Problem detected** | **Number of samples  with 0% API** | **Expiration date of product** | **Type of sector of sample collection** | **Type of outlet of sample collection** | **Place of manufacture** |
| --- | --- | --- | --- | --- | --- | --- | --- | --- | --- | --- | --- | --- |
| Abuga 2013 | Kenya | LMIC | 2006-2010 | 10 | Penicillin injection N=1 Gentamycin injection N=8 Ergometrine N=1 | 1. API 2. Sterility | No problem |  | NI | NI | Central & Peripheral | Imported & National |
| Anyakora 2018 | Nigeria | LMIC | 2016 | 485 | MgSO4 N=160  Misoprostol N=166  Oxytocin N=159 | 1. API 2. Sterility 3. Identification 4. pH 5. Fill volume | API | Misoprostol N=1 | none expired | Private & Public | Central & Peripheral | Imported |
| Dan-ling 2013 (2 phases) | China | UMIC | 2009 & 2011 | 447 | Cefazolin injection N=447 | 1. API 2. Visible foreign matter and insoluble particles 3. Moisture 4. Impurities 5. Cefazolin sodium polymer 6. Identification 7. Clarity of solution | API  Clarity |  | NI | NI | NI | National |
| Guatemala 2015 | Guatemala | LMIC | 2011 | 6 | Oxtytocin N=6 | 1. API 2. Sterility  3. Endotoxin  4. pH 5. Volume in ampoule | No problem |  | none expired | Private & Public | Central & Peripheral | Imported |
| Hagen 2020 | Malawi | LIC | 2017-2018 | 95 | Oxytocin N=65 Misoprostol N=30 | 1. API 2. pH (oxytocin) 3. Dissolution (misoprostol) 4. Identification | API Inadequate dissolution |  | 2 oxytocin, 2 misoprostol | Private & Public | Central & Peripheral | Imported |
| Hall 2016 | 15 countries^2^ | LIC, LMIC, UMIC | 2011 & 2015 | 215 | Misoprostol N=215 | 1. API 2. Appearance 3. Packaging 4. Manufacture date 5. Identification | API Inadequate packaging | Misoprostol N=14 | NI | Private & Public | Central & Peripheral | NI |
| Hozergeil 1993 | Gambia, Malawi, Sudan, Zimbabwe | LiC, LMIC | 1993 | 30 | Ergometrine N=25 Oxytocin N=5 | API | API |  | 4 Ergometrine and 2 oxytocin samples were expired | Public | Central & Peripheral | Imported |
| Islam 2018 | Myanmar | LMIC | 2014 | 58 | Gentamycin injection N=58 | 1. API 2. Sterility 3. Endotoxins 4. Identification 5. Microbial assay | API  Microbial contamination Fake packaging | Gentamycin N=3 | NI | Private & Public | Central & Peripheral | Imported & National |
| Kaale 2016 | Tanzania | LIC | NI (2012 or later) | 15 | Ergometrine N=15 | 1. API 2. Labelling  3. Visual inspection of ampoules | API  Visual inspection |  | NI | Private & Public | Peripheral | NI |
| Karikari-Boateng 2013 | Ghana | LMIC | 2012 | 268 | Ergometrine N=99 Oxytocin N=169 | 1. API 2. Sterility | API Sterility | Oxytocin N=2 | 6 Ergometrine and 14 oxytocin samples without expiration dates | Private & Public | Central & Peripheral | Imported |
| Karwar 2011 | Afghanistan | LIC | 2009 | 35 | Gentamycin injection N=35 | 1. API  2. Identification 3. pH 4. Bacterial endotoxins | No problem |  | none expired (this was an inclusion criterion) | Private & Public | Peripheral | NI |
| Lambert 2018 | DR Congo | LIC | 2016 | 15 | Oxtytocin N=15 | 1. API 2. Labelling and packaging 3. Sterility  4. Degradation products | API Packaging (lack of info on storage temperature) Unknown compound (contaminant?) |  | none expired | Private | Peripheral | Imported |
| Lambert 2019 | Ethiopia | LIC | 2017 | 45 | Oxytocin N=45 | 1. API 2. Sterility 3. Endotoxins | API |  | NI | Private & Public | Central & Peripheral | Imported |
| Liu 2016 | Nepal, Vietnam | LIC, LMIC | NI (2012 or later) | 42 | Oxytocin N=42 | API | API |  | none expired | NI | Peripheral | NI |
| MoPH Afghanistan 2015 | Afghanistan | LIC | 2015 | 57 | Ampicillin powder N=57 | 1. API 2. Identification 3. Packaging | No problem |  | none expired | Public | Central & Peripheral | NI |
| Nazerali 1996 | Zimbabwe | LMIC | 1992 | 168 | Ampicillin injectionN=34  Ergometrine N=93  Penicillin injection N=41 | API | API |  | expired samples were not assessed | Public | Central & Peripheral | National |
| PATH 2015 | India | LMIC | 2014 | 94 | Oxytocin N=94 | 1. API  2. Identification 3. Visual inspection | API |  | none expired | NI | Central & Peripheral | NI |
| Peru 2010 | Peru | UMIC | 2010 | 8 | Oxytocin N=8 | API | No problem |  | none expired | Private & Public | Peripheral | Imported & National |
| Prazuck 2002 | Myanmar | LMIC | 1997 | 2 | Penicillin injection N=2 | 1. API 2. Identification | API |  | 1 expired | Private | Peripheral | Imported |
| Pribluda 2012 | Indonesia | LMIC | 2011 | 110 | Oxytocin N=110 | 1. API 2. Labeling and packaging  3. Contaminant or strange particle matters | API | Oxytocin N=2 | none expired | Public | Central & Peripheral | National |
| Rafiki Islam 2017 | Cambodja | LMIC | 2013 | 59 | Gentamycin injection N=59 | 1. API 2. Identification 3. Sterility 4. Endotoxins  5. Microbial assay | No problem |  | none expired | NI | Central & Peripheral | Imported |
| SAIDI-Peru 2009 | Peru | UMIC | 2008 | 8 | Gentamycin injection N=8 | "All tests in USP Pharmacopeia" | Presence of impurity Wrong insert |  | none expired | Private & Public | Peripheral | Imported & National |
| Scrimgeour 2019 | Papua New Guinea, Vanatu, Solomon Islands | LMIC | 2017-2018 | 90 | Ampicillin injection N=30 Penicillin injection N=30 Metronidazole injection N=30 | API | No problem |  | none expired | NI | Central | NI |
| Sheth 2007 | India | LMIC | NI (2001-2011) | 20 | Gentamycin injection N=20 | 1. API 2. Packaging  3. Physical inspection of formulations | API Fake packaging |  | NI | Private | Peripheral | NI |
| Silva 2010 | Brazil | UMIC | NI (2001-2011) | 13 | Ampicillin injection N=13 | API | No problem |  | none expired | Public | Peripheral | NI |
| Stanton 2012 | Ghana | LMIC | 2010 | 101 | Ergometrine N=55 Oxytocin n=46 | API | API | Ergometrine N=1 | 2 oxytocin samples expired | Private & Public | Peripheral | NI |
| Stanton 2014 | India | LMIC | 2011 | 381 | Ergometrine N=188 Oxytocin n=193 | API | API |  | none expired | Private | Peripheral | National |
| Tabernero 2019 | Laos | LMIC | 2012 | 104 | Ampicillin injection N=104 | 1. API 2. Identification 3. Packaging | API |  | NI | Private | Peripheral | Imported & National |
| Taylor 2001 | Nigeria | LMIC | NI (up to 2000) | 20 | Penicillin injection N=20 | API | API |  | NI | NI | Peripheral | Imported |
| Thoithi 2002 | Kenya | LMIC | 2000 | 7 | Gentamycin powder N=3 Metronidazole injection N=2 Penicillin powder N=2 | 1.API 2. Sterility | API |  | NI | NI | NI | Imported & National |
| Thoithi 2008 | Kenya | LMIC | 2005 | 11 | Ampicillin injection N=2 Cefazolin injection N=2 Gentamycin powder N=3 Metronidazole injection N=2 Penicillin powder N=2 | 1. API 2. Sterility | No problem |  | NI | NI | NI | Imported & National |

| UN Com LSC 2015 | 10 countries^3^ | LIC, LMIC | 2013 | 102 | Ampicillin powder N=26 Gentamycin injection N=29 MgSO4 N=19 Oxytocin N=22 Penicillin powder N=6 | 1. API 2. Related substances 3. pH  4. Extractable volume 5. Appearance | API Related substances Visible particles pH (gentamycin & MgSO4) |  | none expired | Private & Public | Central | Imported & National |
| --- | --- | --- | --- | --- | --- | --- | --- | --- | --- | --- | --- | --- |
| Walker 1988 | BangladeshDR Yemen, Zimbabwe | LIC, LMIC | NI (Before 2001) | 24 | Ergometrine N=24 | API | API |  | 5 samples expired | NI | Peripheral | NI |
| WHO 1995 DAP95.3 | Cameroon, Madgascar, Chad | LIC, LMIC | 1994 | 14 | Penicillin injection N=14 | 1. API 2. Identification | API | Penicillin N=1 | NI | Private & Public | Central & Peripheral | NI |

| 1. When period spanned > 1 year, we considered the last year when samples were collected |  |  |  |  |
| --- | --- | --- | --- | --- |
| 2. Hall 2016, 15 countries: Bangladesh, Egypt, Cambodia, Kenya, India, Mexico, Nigeria, Pakistan, Peru, Vietnam (74 samples). Nigeria, Nepal, Pakistan, Bangladesh, Argentina, Indonesia, Peru, the Philippines, Kasakhstan (141 samples), | | | | |
| 3. UN Com LSC 2015, 10 countries: Burkina Faso, Kenya, Madagascar, Nepal, Nigeria, Tajikistan, Tanzania, Uganda, Vietnam, Zimbabwe | |  |  |  |
