# Supplementary material for: Quality of medicines for life-threatening pregnancy complications in low- and middle-income countries: A systematic review
Source: PLoS One. 2020 Jul 10;15(7):e0236060. doi: 10.1371/journal.pone.0236060 (PMC7351160; doi:10.1371/journal.pone.0236060)
Supplement: S2 Table — (DOCX) [file pone.0236060.s006.docx]

**S2 Table. Quality scores of 34 studies included in the systematic review**

| Study ID | 1.  Timing and location of study cleary stated | 2.  Definition of counterfeit / substandard med provided? | 3. Description of Type of outlet sampled | 4.  Description  of sampling design & sample size calculation | 5.  Type and N of dosage units purchased fr outlet | 6. Random sampling used? | 7.  Info on who collected the sample (mystery shopper)? | 8.  Packaging assessment performed? | 9.  Statistical analysis described? | 10. Chemical analysis clearly described? | 11.  Details  on method validation | 12. Chemical analysis assessors blinded to packaging? | **Total quality score^1^** |
| --- | --- | --- | --- | --- | --- | --- | --- | --- | --- | --- | --- | --- | --- |
| Abuga 2013 | 0 | 0 | 0 | 0 | 0 | 0 | 0 | 0 | 0 | 1 | 0 | 0 | **1** |
| Anyakora 2018 | 1 | 1 | 1 | 1 | 1 | 1 | 1 | 1 | 1 | 1 | 1 | 0 | **11** |
| Dan Ling 2013 | 0 | 0 | 0 | 0 | 0 | 0 | 0 | 0 | 0 | 1 | 0 | 0 | **1** |
| Guatemala 2011 | 1 | 0 | 1 | 0 | 0 | 0 | 0 | 0 | 0 | 1 | 0 | 0 | **3** |
| Hagen  2020 | 1 | 1 | 1 | 1 | 1 | 1 | 1 | 0 | 1 | 1 | 1 | 0 | **10** |
| Hall 2016 | 0 | 1 | 1 | 0 | 0 | 0 | 0 | 1 | 0 | 1 | 0 | 0 | **4** |
| Hozergeil 1993 | 1 | 0 | 1 | 0 | 0 | 0 | 0 | 0 | 0 | 1 | 0 | 1 | **4** |
| Islam 2018 | 0 | 0 | 0 | 0 | 0 | 0 | 0 | 0 | 0 | 1 | 0 | 0 | **1** |
| Kaale 2016 | 0 | 1 | 1 | 0 | 1 | 0 | 0 | 0 | 1 | 1 | 0 | 0 | **5** |
| Karikari 2013 | 1 | 1 | 1 | 0 | 1 | 1 | 1 | 1 | 0 | 1 | 0 | 0 | **8** |
| Karwar 2011 | 1 | 0 | 1 | 0 | 1 | 0 | 0 | 0 | 0 | 1 | 0 | 1 | **5** |
| Lambert 2018 | 1 | 1 | 1 | 0 | 0 | 0 | 1 | 1 | 0 | 1 | 1 | 0 | **7** |
| Lambert 2019 | 1 | 1 | 1 | 0 | 1 | 0 | 1 | 1 | 0 | 1 | 1 | 0 | **8** |
| Liu 2016 | 0 | 0 | 0 | 0 | 1 | 0 | 0 | 0 | 0 | 1 | 0 | 0 | **2** |

| Study ID | 1.  Timing and location of study cleary stated | 2.  Definition of counterfeit / substandard med provided? | 3. Description of Type of outlet sampled | 4.  Description  of sampling design & sample size calculation | 5.  Type and N of dosage units purchased fr outlet | 6. Random sampling used? | 7.  Info on who collected the sample (mystery shopper)? | 8.  Packaging assessment performed? | 9.  Statistical analysis described? | 10. Chemical analysis clearly described? | 11.  Details  on method validation | 12. Chemical analysis assessors blinded to packaging? | **Total quality score^1^** |
| --- | --- | --- | --- | --- | --- | --- | --- | --- | --- | --- | --- | --- | --- |
| MoPH Afghanistan 2015, | 1 | 1 | 0 | 0 | 0 | 1 | 0 | 1 | 0 | 0 | 0 | 0 | **4** |
| Nazerali 1996 | 0 | 1 | 1 | 0 | 1 | 0 | 0 | 0 | 1 |  | 1 | 0 | **5** |
| PATH 2015 | 1 | 1 | 1 | 1 | 1 | 1 | 0 | 0 | 0 | 1 | 0 | 0 | **7** |
| Peru 2010 | 1 | 0 | 1 | 0 | 0 | 0 | 0 | 0 | 0 | 0 | 0 | 0 | **2** |
| Prazuck 2002 | 0 | 1 | 1 | 0 | 0 | 0 | 0 | 0 | 0 | 1 | 0 | 0 | **3** |
| Pribluda 2012 | 1 | 0 | 1 | 1 | 0 | 1 | 0 | 1 | 0 | 1 | 0 | 1 | **7** |
| Rafiki Islam 2017 | 1 | 0 | 1 | 0 | 1 | 0 | 0 | 1 | 0 | 0 | 0 | 0 | **4** |
| SAIDI-Peru 2009 | 0 | 0 | 1 | 0 | 1 | 0 | 0 | 1 | 0 | 1 | 0 | 0 | **4** |
| Scrimgeour 2019 | 0 | 1 | 1 | 1 | 1 | 0 | 0 | 1 | 0 | 1 | 0 | 1 | **7** |
| Sheth 2007 | 0 | 1 | 1 | 1 | 1 | 1 | 1 | 1 | 0 |  | 0 |  | **7** |
| Silva 2010 | 0 | 1 | 1 | 0 | 1 | 0 | 0 | 0 | 0 | 1 | 1 | 0 | **5** |
| Stanton 2012 | 1 | 1 | 1 | 1 | 0 | 1 | 1 | 1 | 0 | 1 | 0 | 0 | **8** |
| Stanton 2014 | 1 | 1 | 1 | 1 | 0 | 1 | 1 | 1 | 1 | 1 | 0 | 0 | **9** |
| Tabernero 2019 | 1 | 1 | 1 | 1 | 1 | 1 | 1 | 1 | 0 | 1 | 0 | 1 | **10** |
| Taylor 2001 | 0 | 1 | 1 | 0 | 0 | 0 | 1 | 0 | 0 | 1 | 1 | 0 | **5** |
| Thoithi 2002 | 0 | 0 | 0 | 0 | 0 | 0 | 0 | 0 | 0 | 1 | 0 | 0 | **1** |
| Thoithi 2008 | 0 | 0 | 0 | 0 | 0 | 0 | 0 | 0 | 0 | 1 | 0 | 0 | **1** |
| U.N Col 2015 | 1 | 1 | 1 | 0 | 1 | 0 | 0 | 0 | 0 | 1 | 0 | 0 | **5** |
| Walker 1988 | 0 | 1 | 1 | 0 | 0 | 0 | 0 | 0 | 0 | 1 | 0 | 0 | **3** |
| WHO 1995 | 0 | 1 | 0 | 0 | 0 | 0 | 1 | 0 | 0 | 0 | 0 | 1 | **3** |
| **TOTAL** | **17** | **21** | **26** | **9** | **16** | **10** | **11** | **14** | **5** | **28** | **7** | **6** |  |

Based on MEDQUARG guidelines 12 Quality Criteria (Newton 2009, Almuzaini 2013)
