# Supplementary material for: Quality of medicines for life-threatening pregnancy complications in low- and middle-income countries: A systematic review
Source: PLoS One. 2020 Jul 10;15(7):e0236060. doi: 10.1371/journal.pone.0236060 (PMC7351160; doi:10.1371/journal.pone.0236060)
Supplement: S3 Table — (DOCX) [file pone.0236060.s007.docx]

**S3 Table. Prevalence of failed samples over time**

| **Medicine** | **Year of sample collection** | | | P Value | **Total** |
| --- | --- | --- | --- | --- | --- |
|  | **up to 2000** | **2001-2011** | **2012 and later** |  |  |
| **Oxytocin** |  |  |  |  |  |
| N studies | 1 | 5 | 8 |  | 14 |
| Total N samples | 5 | 363 | 611 |  | 979 |
| N failed samples | 4 | 114 | 271 |  | 389 |
| % of failed samples | 80.0 | 31.4 | 44.4 | <0.001^1^ | 39.7 |
| **Ergometrine** |  |  |  |  |  |
| N studies | 3 | 3 | 2 |  | 8 |
| Total N samples | 142 | 244 | 114 |  | 500 |
| N failed samples | 99 | 190 | 88 |  | 377 |
| % of failed samples | 69.7 | 77.9 | 77.2 | 0.176^2^ | 75.4 |
| **Misoprostol** |  |  |  |  |  |
| N studies | 0 | 0 | 3 |  | 3 |
| Total N samples | 0 | 0 | 411 |  | 411 |
| N failed samples | 0 | 0 | 159 |  | 159 |
| % of failed samples | 0 | 0 | 38.7 | NA | 38.7 |
| **All Uterotonics** |  |  |  |  |  |
| N studies | 4 | 8 | 13 |  | 25 |
| Total N samples | 147 | 607 | 1136 |  | 1890 |
| N failed samples | 103 | 304 | 518 |  | 925 |
| % of failed samples | 70.1 | 50.1 | 45.6 | <0.001^2^ | 48.9 |

1. Fischer´s exact test. difference between first versus the other two periods. 2. Pearson´s Chi squared test.

NA. Not applicable

| **Magnesium sulphate** | **up to 2000** | **2001-2011** | **2012 and later** | P Value | **Total** |
| --- | --- | --- | --- | --- | --- |
| N studies | 0 | 0 | 2 |  | 2 |
| Total N samples | - | - | 179 |  | 179 |
| N failed samples | - | - | 6 |  | 6 |
| % of failed samples | - | - | 3.4 | NA | 3.4 |

NA. Not applicable

| **Medicine** | **Year of sample collection** | | | P Value | **Total** |
| --- | --- | --- | --- | --- | --- |
|  | **up to 2000** | **2001-2011** | **2012 and later** |  |  |
| **Ampicillin** |  |  |  |  |  |
| N studies | 1 | 2 | 4 |  | 7 |
| Total N samples | 34 | 15 | 217 |  | 266 |
| N failed samples | 7 | 0 | 29 |  | 36 |
| % of failed samples | 20.6 | 0.0 | 13.4 | 0.146^1^ | 13.5 |
| **Cephazolin** |  |  |  |  |  |
| N studies | 0 | 2 | 0 |  | 2 |
| Total N samples | 0 | 449 | 0 |  | 449 |
| N failed samples | 0 | 72 | 0 |  | 72 |
| % of failed samples | 0 | 16.0 | 0 | NA | 16.0 |
| **Gentamycin** |  |  |  |  |  |
| N studies | 1 | 5 | 3 |  | 9 |
| Total N samples | 3 | 74 | 146 |  | 223 |
| N failed samples | 1 | 5 | 15 |  | 21 |
| % of failed samples | 33.3 | 6.8 | 10.3 | 0.197^1^ | 9.4 |
| **Penicillin G** |  |  |  |  |  |
| N studies | 5 | 2 | 2 |  | 9 |
| Total N samples | 79 | 3 | 36 |  | 118 |
| N failed samples | 16 | 0 | 0 |  | 16 |
| % of failed samples | 20.3 | 0.0 | 0.0 | 0.006^1^ | 13.6 |
| **Metronidazole** |  |  |  |  |  |
| N studies | 1 | 1 | 1 |  | 3 |
| Total N samples | 2 | 2 | 30 |  | 34 |
| N failed samples | 1 | 0 | 0 |  | 1 |
| % of failed samples | 50.0 | 0.0 | 0.0 | 0.118^1^ | 2.9 |
| **All Antibiotics** |  |  |  |  |  |
| N studies | 8 | 12 | 10 |  | 30 |
| Total N samples | 118 | 543 | 429 |  | 1090 |
| N failed samples | 25 | 77 | 44 |  | 146 |
| % of failed samples | 21.2 | 14.2 | 10.3 | 0.006^2^ | 13.4 |

1. Fischer´s exact test. 2. Pearson´s Chi squared test.

NA. Not applicable
