# Supplementary material for: Quality of medicines for life-threatening pregnancy complications in low- and middle-income countries: A systematic review
Source: PLoS One. 2020 Jul 10;15(7):e0236060. doi: 10.1371/journal.pone.0236060 (PMC7351160; doi:10.1371/journal.pone.0236060)
Supplement: S4 Table — (DOCX) [file pone.0236060.s008.docx]

**S4 Table. Failed samples due to inadequate active pharmaceutical ingredient (API)**

| **Study** | **Country** | **Total N samples tested** | **N failed samples** | **% Failed samples** | **Inadequate API in general** | | **Low API** | | **Observations on samples with low API** |
| --- | --- | --- | --- | --- | --- | --- | --- | --- | --- |
|  |  | N | n | % | n | (%) | n | (%) |  |
| **Oxtytocin** |  |  |  |  |  |  |  |  |  |
| Stanton 2012 | Ghana | 46 | 35 | (76.1) | 35 | (76.1) | 35 | (76.1) | 35 total low fails: 12 had 1-39% API, 4 had 40-59% API, 19 had 60-89% API |
| Karikari 2013 | Ghana | 169 | 94 | (55.6) | 94 | (55.6) | 94 | (55.6) | 94 total low fails: 2 had 0% API. No details on other samples. |
| Stanton 2014 | India | 193 | 69 | (35.8) | 69 | (35.8) | 57 | (29.5) | 57 total low fails: 5 had 0-5% API, 4 had 6-50% API, 12 had 51-75% API, 36 had 76-89% API. |
| Hogerzeil 1993 | Zimbabwe | 5 | 4 | (80.0) | 4 | (80.0) | 0 | (0.0) | NA |
| Pribluda 2012 | Indonesia | 110 | 10 | (9.1) | 10 | (9.1) | 10 | (9.1) | 10 total low fails: 2 had 0% API. No other details. |
| MQ Database 2011 | Guatemala | 6 | 0 | (0.0) | 0 |  |  |  | NA |
| MQ Database 2010 | Peru | 8 | 0 | (0.0) | 0 |  |  |  | NA |
| UN Col LSC  2015 | 10 countries | 22 | 8 | (36.4) | 8 | (36.4) | 8 | (36.4) | 8 total low fails: 2 had 51-75% API, 6 had 76-89% API. |
| Anyakora 2018 | Nigeria | 159 | 118 | (74.2) | 118 | (74.2) | 115 | (72.3) | No details on samples with low API. |
| Lambert 2018 | Democratic Republic of Congo | 15 | 12 | (80.0) | 12 | (80.0) | 12 | (80.0) | 12 total low fails: 4 had 51-75% API, 8 had 76-89% API. |
| PATH  2015 | India | 94 | 14 | (14.9) | 14 | (14.9) | 11 | (11.7) | 11 total low fails 1 had 6-50% (45.9%) API, 1 had 51-75% API, 9 had 76-89% API. |
| Lambert 2019 | Ethiopia | 45 | 2 | (4.4) | 2 | (4.4) | 2 | (4.4) | 2 total low fails: 2 had 76-89% API |
| Liu  2016 | Nepal, Vietnam | 42 | 13 | (31.0) | 13 | (31.0) | NI |  | NA |
| Hagen  2020 | Malawi | 65 | 10 | (15.4) | 10 | (15.4) | 10 | (15.4) | 10 total low fails: range 82% to 89% |
| **TOTAL** |  | **979** | **389** | **(39.7)** | **389** | **(39.7)** | **354** | **(36.2)** |  |
| **Ergometrine** |  |  |  |  |  |  |  |  |  |
| Stanton 2012 | Ghana | 55 | 55 | (100.0) | 55 | (100.0) | 54 | (98.2) | 54 total low fails: 1 had 0% API, 13 had 1-39% API, 28 had 40-59% API, 12 had 60-89% API. |
| Karikari 2013 | Ghana | 99 | 73 | (73.7) | 73 | (73.7) | 73 | (73.7) | 73 total low fails: 0 had 0% API. No other details. |
| Stanton 2014 | India | 188 | 135 | (71.8) | 135 | (71.8) | 135 | (71.8) | 135 total low fails: 2 had 0-25%, 5 had 26-50%, 16 had 51-75%, 112 had 76-89% |
| Hozergeil 1993 | Malawi, Gambia, Sudan, Zimbabwe | 25 | 19 | (76.0) | 19 | (76.0) | 19 | (76.0) | 19 total low fails: 8 had < 60% API, 7 had 60-79% API, 4 had 80-89% API. |
| Kaale  2016 | Tanzania | 15 | 15 | (100.0) | 15 | (100.0) | 15 | (100.0) | 15 total low fails: 1 had 0-25% API (16% API), 4 had 26-50% API, 9 had 51-75% API, 1 had 76-89% |
| Walker  1988 | Bangladesh, DR Yemen, Zimbabwe | 24 | 15 | (62.5) | 15 | (62.5) | 15 | (62.5) | 15 total low fails: 8 had 80-89% API, 7 had < 20% API but none had 0% API. |
| Nazerali 1996 | Zimbabwe | 93 | 65 | (69.9) | 65 | (69.9) | 65 | (69.9) | Low fail samples: The 95% CI of API is 57-89%. No other details on samples with low API. |
| Abuga  2013 | Kenya | 1 | 0 | (0.0) | 0 | (0.0) | 0 | (0.0) | NA |
| **TOTAL** |  | **500** | **377** | **(75.4)** | **377** | **(75.4)** | **376** | **(75.2)** |  |

| **Study** | **Country** | **Total N samples tested** | **N failed samples** | **% Failed samples** | **Inadequate API** | | **Low API** | | **Observations on samples with low API** |
| --- | --- | --- | --- | --- | --- | --- | --- | --- | --- |
|  |  | N | n | (%) | n | (%) | n | (%) |  |
| **Misoprostol** |  |  |  |  |  |  |  |  |  |
| Anyakora 2018 | Nigeria | 166 | 56 | (33.7) | 56 | (33.7) | 56 | (33.7) | 56 total low fails: 1 had 0% API. No other details. |
| Hall  2016 | 15 countries^4^ | 215 | 96 | (44.7) | 96 | (44.7) | 85 | (39.5) | 85 total low fails: 14 had 0% API. No other details. |
| Hagen  2020 | Malawi | 30 | 7 | (23.3) | 7 | (23.3) | 7 | (23.3) | 7 total low fails ranging from 12.7% to 53.0% API. 3 samples had 0-25% API, 3 had 26-50% API, and 1 had 51-75% API |
| **Total** |  | **411** | **159** | **(38.7)** | **159** | **(38.7)** | **148** | **(36.0)** |  |

| **MgSO4** |  |  |  |  |  |  |  |  |  |
| --- | --- | --- | --- | --- | --- | --- | --- | --- | --- |
| Anyakora 2018 | Nigeria | 160 | 4 | (2.5) | 4 | (2.5) | NI | NI | NA |
| UNCol 2015 | 10 countries^3^ | 19 | 2 | (10.5) | 0 |  |  |  | NA |
| **TOTAL** |  | **179** | **6** | **(3.4)** | **4** | **(2.,2)** |  |  |  |

| **Study** | **Country** | **Total N samples tested** | **N failed samples** | **% Failed samples** | **Inadequate API** | | **Low API** | | **Observations on samples with low API** |
| --- | --- | --- | --- | --- | --- | --- | --- | --- | --- |
|  |  | N | n | (%) | n | (%) | n | (%) |  |
| **Ampicillin** |  |  |  |  |  |  |  |  |  |
| UnCol 2015 | 10 countries^3^ | 26 | 9 | (34.6) | 3 | (11.5) | 3 | (11.5) | 3 total low fails (< 95% accepted lower limit): 93.3%, 93.9% and 94.3% API. |
| Silva 2010 | Brazil | 13 | 0 | (0.0) | 0 |  | 0 |  | NA |
| Nazerali 1996 | Zimbabwe | 34 | 7 | (20.6) | 7 | (20.6) | 7 | (20.6) | 7 total low fails. No other details. |
| Tabernero 2019 | Laos | 104 | 20 | (19.2) | 20 | (19.2) | NI | NI | NA |
| Thoithi 2008 | Kenya | 2 | 0 | (0.0) | 0 |  |  |  | NA |
| Afghanistan 2015 | Afghanistan | 57 | 0 | (0.0) | 0 |  |  |  | NA |
| Scrimgeour 2019 | Papua New Guinea, Vanatu, Solomon Islands | 30 | 0 | (0,0) | 0 |  |  |  | NA |
| **TOTAL** |  | **266** | **36** | **(13.5)** | **30** | **(11.3)** | **10** | **(3.8)** |  |

| **Study** | **Country** | **Total N samples tested** | **N failed samples** | **% Failed samples** | **Inadequate API** | | **Low API** | | **Observations on samples with low API** |
| --- | --- | --- | --- | --- | --- | --- | --- | --- | --- |
|  |  | N | n | (%) | n | (%) | n | (%) |  |
| **Cefazolin** |  |  |  |  |  |  |  |  |  |
| Dan Ling 2013 | China | 447 | 72 | (16.1) | 2 | (0.8) | 2 | (0.8) | 2 total low fails: No other details. |
| Thoithi 2008 | Kenya | 2 | 0 | (0.0) | 0 |  |  |  | NA |
| **TOTAL** |  | **449** | **72** | **(16.0)** | **2** | **(0.4)** | **2** | **(0.4)** |  |
| **Gentamycin** |  |  |  |  |  |  |  |  |  |
| UnCol 2015 | 10 countries^3^ | 29 | 12 | (41.4) | 6 | (20.7) | 5 | (17.2) | 5 total low fails (< 95% accepted lower limit): 87.8 – 95.3% API. |
| Islam 2018 | Myanmar | 58 | 3 | (5.2) | 3 | (5.2) | 3 | (5.2) | 3 total low fails: all 3 had 0% API |
| Rafiqul Islam 2017 | Cambodja | 59 | 0 | (0.0) | 0 |  |  |  | NA |
| Sheth 2007 | India | 20 | 2 | (10.0) | 2 | (10.0) | 2 | (10.0) | 2 total low fails: both had 80% API |
| Thoithi 2008 | Kenya | 3 | 0 | (0.0) | 0 |  |  |  | NA |
| Thoithi 2002 | Kenya | 3 | 1 | (33.3) | 1 | (33.3) | NI |  | NA |
| Abuga 2013 | Kenya | 8 | 0 | (0.0) | 0 |  |  |  | NA |
| Karwar 2011 | Afghanistan | 35 | 0 | (0.0) | 0 |  |  |  | NA |
| SAIDI-Peru 2009 | Peru | 8 | 3 | (37.5) | 0 |  |  |  | NA |
| **TOTAL** |  | **223** | **21** | **(9.4)** | **12** | **(5.4)** | **10** | **(4.5)** |  |

| **Study** | **Country** | **Total N samples tested** | **N failed samples** | **% Failed samples** | **Inadequate API** | | **Low API** | | **Observations on samples with low API** |
| --- | --- | --- | --- | --- | --- | --- | --- | --- | --- |
|  |  | N | n | (%) | n | (%) | n | (%) |  |
| **Metronidazole** |  |  |  |  |  |  |  |  |  |
| Thoithi 2008 | Kenya | 2 | 0 | (0.0) | 0 |  |  |  | NA |
| Thoithi 2002 | Kenya | 2 | 1 | (50.0) | 1 | (50.0) | NI |  | NA |
| Scrimgeour 2019 | Papua New Guinea, Vanatu, Solomon Islands | 30 | 0 | (0.0) | 0 |  |  |  | NA |
| **TOTAL** |  | **34** | **1** | **(2.9)** | **1** | **(2.9)** | **NI** |  |  |
| **Penicillin G** |  |  |  |  |  |  |  |  |  |
| UnCol 2015 | 10 countries^3^ | 6 | 0 | (0.0) | 0 |  |  |  | NA |
| Taylor 2001 | Nigeria | 20 | 11 | (55.0) | 11 | (55.0) | NI |  | NA |
| Prazuck 2002 | Myanmar | 2 | 1 | (50.0) | 1 | (50.0) | 1 | (50.0) | 1 total low fails: had 45% API |
| WHO 1995 | Cameroon, Madagascar,Tchad | 14 | 2 | (14.3) | 2 | (14.3) | NI |  | NA |
| Nazerali 1996 | Zimbabwe | 41 | 1 | (2.4) | 1 | (2.4) | 0 |  | NA |
| Thoithi 2008* | Kenya | 2 | 0 | (0.0) | 0 |  |  |  | NA |
| Thoithi 2002 | Kenya | 2 | 1 | (50.0) | 1 | (50.0) | NI |  | NA |
| Abuga 2013 | Kenya | 1 | 0 | (0.0) | 0 |  |  |  | NA |
| Scrimgeour 2019 | Papua New Guinea, Vanatu, Solomon Islands | 30 | 0 | (0.0) | 0 |  |  |  | NA |
| **TOTAL** |  | **118** | **16** | **(13.6)** | **16** | **(13.6)** | **1** | **(0.8)** |  |

NA: Not applicable
